# ‘That’s how we got around it’: a qualitative exploration of healthcare professionals’ experiences of care provision for asylum applicants’ with limited English proficiency in UK contingency accommodation

**DOI:** 10.1136/bmjopen-2023-074824

**Published:** 2023-11-23

**Authors:** Louise Tomkow, Gabrielle Prager, Jessica Drinkwater, Rebecca L Morris, Rebecca Farrington

**Affiliations:** 1 The University of Manchester, Manchester, UK; 2 Johns Hopkins University, Baltimore, Maryland, USA; 3 Centre for Primary Care and Health Services Research, The University of Manchester, Manchester, UK; 4 Centre for Primary Care, The University of Manchester, Manchester, UK; 5 Division of Medical Education, University of Manchester Faculty of Biology, Medicine and Health, Manchester, UK

**Keywords:** health services accessibility, primary health care, health equity, quality in health care, organisation of health services

## Abstract

**Objectives:**

The inadequate provision of language interpretation for people with limited English proficiency (LEP) is a determinant of poor health, yet interpreters are underused. This research explores the experiences of National Health Service (NHS) staff providing primary care for people seeking asylum, housed in contingency accommodation during COVID-19. This group often have LEP and face multiple additional barriers to healthcare access. Language discrimination is used as a theoretical framework. The potential utility of this concept is explored as a way of understanding and addressing inequities in care.

**Design:**

Qualitative research using semistructured interviews and inductive thematic analysis.

**Setting:**

An NHS primary care service for people seeking asylum based in contingency accommodation during COVID-19 housing superdiverse residents speaking a wide spectrum of languages.

**Participants:**

Ten staff including doctors, nurses, mental health practitioners, healthcare assistants and students participated in semistructured online interviews. Some staff were redeployed to this work due to the pandemic.

**Results:**

All interviewees described patients’ LEP as significant. Inadequate provision of interpretation services impacted the staff’s ability to provide care and compromised patient safety. Discrimination, such as that based on migration status, was recognised and challenged by staff. However, inequity based on language was not articulated as discrimination. Instead, insufficient and substandard interpretation was accepted as the status quo and workarounds used, such as gesticulating or translation phone apps. The theoretical lens of language discrimination shows how this propagates existing social hierarchies and further disadvantages those with LEP.

**Conclusions:**

This research provides empirical evidence of how the inadequate provision of interpreters forces the hand of healthcare staff to use shortcuts. Although this innovative ‘tinkering’ allows staff to get the job done, it risks normalising structural gaps in care provision for people with LEP. Policy-makers must rethink their approach to interpretation provision which prioritises costs over quality. We assert that the concept of language discrimination is a valuable framework for clinicians to better identify and articulate unfair treatment on the grounds of LEP.

**STRENGTHS AND LIMITATIONS OF THIS STUDY**

- There is no existing research exploring UK healthcare professionals’ experiences of working with asylum seekers with limited English proficiency.
- We are the first to use the theoretical framework of language discrimination in a UK healthcare setting and show how it might be of value in improving care.
- The qualitative data collected is in depth and rich.
- The sample size is modest, due to the specialist nature of the service.
- Our research design stakeholder group was made up of healthcare professionals. Future research in this area should involve patient and public contributors.

## Introduction

Good-quality communication between healthcare providers and patients is crucial for universal access to safe person-centred care.^1^ In English-speaking countries worldwide, including Australia, USA and the UK, patients with limited English proficiency (LEP) experience barriers accessing care,^2 3^ more medical errors,^4 5^ lower satisfaction^6^ and misunderstanding of health information.^7^ The benefits of providing high-quality interpretation include: improved clinical care,^8 9^ reduced inequalities in healthcare access^10^ and cost savings through decreased hospital readmissions and length of stay.^11 12^ Despite this, interpretation services remain underused.^13^ This is justified by time constraints, lack of availability of interpreters and dissatisfaction with interpretation quality.^13^ Worryingly, a study exploring doctors’ reasoning suggested that a clinician-centred approach to communication might be contributory—for some doctors, communication was seen merely as a way of collecting information.^14^

Addressing population health inequalities is touted as a priority for the UK’s National Health Service (NHS) and is foregrounded in pandemic recovery policy.^15 16^ NHS England outlines key principles for the provision of high-quality interpretation and translation services.^17^ However, a recent UK review identified that long-standing ethnic inequalities in access, experience and healthcare outcomes are due, in part, to a lack of appropriate interpretation services.^18^ Despite care providers’ duty to provide interpretation services, research shows this is highly variable in practice according to service users. Family members are often used to fill the gap, with implications for patients’ confidentiality.^2 17 19 20^ Although we know the harmful effects of poor communication on care outcomes, there is scarce research focusing on clinicians’ experiences and negotiation of language barriers.

### Research context

This paper examines the experiences of NHS staff working with people with LEP in UK asylum contingency accommodation during the COVID-19 pandemic. This accommodation housed people seeking asylum from multiple countries, that is, engaged in the lengthy legal process of applying for refugee status. A refugee is a person who ‘owing to a well-founded fear of being persecuted for reasons of race, religion, nationality, membership of a particular social group or political opinion, is outside the country of his nationality, and is unable to or, owing to such fear, is unwilling to avail himself of the protection of that country’.^21^ While awaiting a decision on refugee status individuals are usually housed in dispersal accommodation. Contingency accommodation—such as hotels or army barracks—is used for extra capacity. Over the last decade, the UK government introduced policies purposefully making life difficult for migrants to reduce numbers. These policies and practices, often described as the ‘hostile environment’, embed immigration controls into public services.^22^

People seeking asylum face multiple obstacles accessing healthcare and frequently have diverse and additional needs to settled populations.^2^ Immigration controls present in UK welfare and healthcare systems include restrictions to entitlements for free NHS care. Since 2017, certain migrants are ineligible for non-emergency care and must pay before receiving treatment. Although GP services remain free for all, there is confusion among NHS care providers about charging and people seeking asylum often lack awareness of how to navigate the NHS.^23^ Inadequate interpretation is a significant barrier to care.^24^ The time-limited structure of NHS consultations can be inadequate to address complex needs.^25^

The use of contingency accommodation increased considerably in the COVID-19 pandemic,^26^ amplifying the difficulties of healthcare access and living conditions. Prepandemic healthcare access for people seeking asylum housed this way was documented as inadequate; evidence suggests this has deteriorated significantly and concerns continue to be raised regarding unsafe conditions.^27^ During the pandemic, many NHS services adopted remote working, worsening existing barriers to access and communication.^28^ In recognition, some primary care providers established extra services focusing on health needs in contingency accommodation.^29^ This paper examines NHS staff experiences at one such service, rapidly implemented in 2020, delivering face-to-face primary care on-site for over 200 people seeking asylum. Many of the staff were redeployed as part of the COVID-19 response. Interpretation for superdiverse residents who spoke a multitude of languages was from a mix of untrained informal contacts, community volunteers, bilingual practitioners and trained professional on-demand telephone interpreters. It was of variable quality and availability.

### Research aims

This research aims to investigate the experiences of NHS staff during COVID-19, examining how staff understood, experienced and negotiated care provision for people seeking asylum with LEP. As a secondary aim, we explore the potential utility of the concept of language discrimination as a way of comprehending inequities in access to care.

### Theoretical framework

We use the concept of language discrimination as a theoretical framework.^30^ We understand this concept as articulated by Lippi-Green: a sociological theoretical lens to observe how discrimination on the grounds of LEP perpetuates inequitable treatment and reinforces normative power hierarchies. We do so from an antiracist position that embraces naming discrimination, then asking ‘how is it operating here?’ as the first steps to addressing it.^31^

Lippi-Green’s work examines how discrimination on the grounds of accent or LEP benefits those who speak English as a first language over those with LEP.^30^ The US studies have shown how language discrimination causes individuals to feel overlooked and inferior^32^ and is associated with adverse health outcomes.^33^ There is little exploration of how it operates beyond the USA. In UK healthcare, this scarcity of work describing language discrimination is perhaps related to opacity over the legal protection offered to those with LEP. It is not considered a ‘protected characteristic’ under the Equality Act 2010. However, both socially and legally, language is inextricably intertwined with nationality and race, which are protected characteristics.^33–36^ As a result, NHS England’s interpretation commissioning guidance acknowledges discrimination on the basis of national origin through a lack of language assistance for LEP persons.^17^ Consequently, we view it as an essential strategic consideration in reducing health inequalities.

We conceptualise language discrimination as both structural—created and normalised in mainstream institutions—and structuring—in that it reinforces existing hierarchies of privilege, restricting the opportunities of already-disadvantaged groups. The inadequate provision of interpretation services epitomises the operationalisation of language discrimination in healthcare and is entangled in the UK context of a hostile environment. It is important to note that adequate provision of interpreters alone would not eradicate language discrimination, as cultural humility remains important. However, for the purpose of this paper, we use adequate interpretation provision as the bare minimum, thus a proxy marker of language discrimination, and as an empirical signal to explore the issue.

## Methods

### Study design

Twenty-seven healthcare professionals currently or previously working with the asylum seeker primary care service in contingency accommodation were invited via email to participate in an online interview about their experiences. In 2021, 10 of these healthcare professionals participated in remote semistructured qualitative interviews, lasting between 45 min and 2.5 hours. Participants included doctors, nurses, mental health practitioners, healthcare assistants and students, some of whom had been redeployed to primary care in the pandemic. Interviews were conducted using Zoom video conferencing software by author GP, a clinical academic with no prior relationship to the invited healthcare professionals. Interviews began with verbal consent and participants shared a brief explanation of their professional background and role. Participants were told that the research team were interested in migrant health and their experiences working at the service during COVID-19. Interviews were conversational, with open questioning using an interview guide on the practical and ethical issues of care provision in contingency accommodation. They allowed opportunities for participants to discuss issues and experiences important to them. The interview guide was developed in collaboration with health professionals experienced in migrant health work. Audio of the interviews was recorded, notes taken by the interviewer, and recordings transcribed verbatim by GP. No payment was offered for participation. Data were anonymised, with place names changed and pseudonyms to protect participants. Demographic details for individual participants are not shared due to the modest number of participants, the specialist nature of the service, and to maintain confidentiality. Two site visits were undertaken by researchers (LT and GP) to familiarise with the environment.

### Data analysis

Using inductive thematic analysis, LT and GP coded data using NVivo V.12 to increase robustness.^33^ Dominant themes were derived from the transcripts before data were coded and extracted. First phase analysis focused on the moral implications of care in contingency accommodation and is explored elsewhere.^37^ Language and discrimination appeared as key themes and prompted additional examination. Secondary thematic analysis was, therefore, applied to the data using Lippi-Green’s language discrimination as a theoretical framework (by GP, LT, JD, RLM and RF). This occurred after data collection as specific questions about language and discrimination were not in the interview topic guide.

### Research team and reflexivity

The research team are clinical academics with an interest in migrant health. LT (PI) and RF designed and led the research. LT developed study materials and ethics application. LT supervised GP in recruitment and interviews. All authors (LT, GP, JD, RLM and RF) contributed to the analysis. Our interest in migrant health risks the imposition of our own beliefs on the research. LT and RF work in migrant health advocacy which facilitated access to the participants but could also shape both the data interpretation and the ideas in this paper. The clinical identity of the interviewer (GP) may have promoted a social desirability bias from participants. In mitigation, the interview topic guide was developed with several clinicians.

### Patient and public involvement

Patients and/or the public were not involved in the design, or conduct, or reporting, or dissemination plans of this research.

## Results

All participants stressed the importance of patients’ LEP and the inadequate provision of interpretation services on care provision. Many commented on how patients were disadvantaged on the grounds of language and how this compromised patient safety. However, unlike other forms of discrimination such as racism, the disadvantage faced by people with LEP was rarely described in terms of discrimination. Instead, staff responded by improvising, using workarounds to get the job done. To illustrate, we present three emergent themes from the data: (1) recognising and resisting discrimination; (2) the importance of interpretation provision for safe care and (3) improvisation around inadequate interpretation.

### Recognising and resisting discrimination

Healthcare staff readily recognised that people seeking asylum encountered discrimination on the grounds of migration status, nationality and race:

They will face a lot of discrimination in the quality [of care] … It’s been a rude awakening … there’s a lot of prejudice and stereotypes of those seeking asylum. Participant 7

Many noticed discrimination towards patients from healthcare staff in other settings such as pharmacy, hospital and social care. Staff reported inappropriate questioning of patients’ entitlement to NHS treatments and social care by service providers, resulting in some being denied care which they had the right to receive. Several portrayed how a dental surgery had refused to see residents from contingency accommodation and labelled this as discriminatory. One participant used these allegations of discrimination as a way of challenging dental surgery staff and advocating for all the residents:

I said, “Can I just get this straight? So, you’re telling me that one individual was abusive towards your staff, and because of that, you’re going to be discriminating against 250 other individuals because you’re deciding that everybody who is seeking asylum must be the same and is going to behave like this one person. Is that what you’re saying?” … “No, no, no. That’s not what we’re saying.” “Okay, good. Because that would have been discrimination, wouldn’t it.” These are the conversations that we have to have all of the time. Participant 1

This illustrates how recognising inequity as discrimination—and articulating it as such—allows individuals to challenge and resist inequitable treatment. Importantly, it demonstrates that some healthcare staff also recognise calling out discrimination as part of their role.

### The importance of interpretation provision for safe care

When asked about wider experiences working in the contingency accommodation, all participants spontaneously stressed the importance of LEP and contrasted it with their previous NHS work. They acknowledged how LEP compounded the multiple complex needs of their patients and the importance of language interpretation services to reduce inequity:

I mean, in my view, [translation services] should be a bare minimum for any service delivering health care in the UK … Because otherwise, you create instant inequalities. Particularly within this very, very vulnerable group of people. Why that isn’t a national standard? I don’t know. Participant 1

It’s quite chaotic … The difference, obviously the patient group have a lot of needs that need to be addressed and to be managed, especially the fact that English is not their first language. Participant 7

Participants frequently associated inadequate interpretation provision with care inequities, even highlighting safety concerns, such as COVID-19 transmission risk. This illustrates the wider public health implications of the derisory provision of information to patients with LEP:

In the really early days when they were not providing information about COVID in their own languages, a lot of them didn’t know what COVID was. Participant 10

Participant 10 draws attention to the risks posed by medication errors:

The patients here didn’t speak English. They can’t read road signs, they don’t know where the GP practice is, or the pharmacy is. And when they get to the pharmacy, they can’t communicate with the lady behind the desk, so it was really difficult in that sense, and then they would get the medication and be like, “I can’t read the label. How do I take this?” And then there are safety aspects to that in that they are not ingesting their medication in the right way and things like that, so that was difficult, so things that are normally easy are really, really hard. Participant 10

This illustrates multiple structural barriers faced by patients with LEP trying to navigate health systems, access care and self-manage. It demonstrates the need for improvements in interpretation services beyond medical consultations, including translation of signage, pamphlets and labels. LEP instigates barriers to accessing safe care and achieving good health not encountered by English speakers, showing how language discrimination propagates existing social hierarchies. Although participants recognised structured and structuring aspects of inadequate interpretation provision, they did not articulate it as discriminatory.

### Improvisation around inadequate interpretation

All participants reported facing challenges accessing adequate interpretation services. Many described the resulting suboptimal care. As part of the pandemic response, volunteers from local refugee charities initially provided face-to-face interpretation at the contingency accommodation for medical consultations. This in-person service was described by participant 2 as ‘*so useful … made life so much easier … made such a difference’*. However, this was impossible beyond the first few months as their own voluntary organisations reopened. The clinical team were left using telephone interpretation services, universally reported as problematic and often labelled a ‘waste’ of time:

The waits for [the telephone interpretation service] were quite extensive. 30 minutes, 45 minutes, which had a huge impact on clinical time. And you would often get through and then not be able to get through to the patient. We were wasting a huge number of clinical hours at that point in just trying to get hold of patients and speak to them. Participant 1

The poor mobile phone signal in the accommodation caused frequent and lengthy interruptions. When staff were able to connect, the quality of interpretation was variable and often substandard:

Someone would talk at length sometimes and the interpreter would summarise it in a few sentences, so I don’t know if I was actually getting the full picture … sometimes because [patients] had partial English they would sometimes say ‘I’m not saying that’. Participant 9

Inadequate interpretation services in secondary care impacted the workload of the primary care participants. Staff in an already-pressured environment took time to fill the gap left by inadequate language provision:

The doctor sends the referrals, and then the patient gets a letter, but they can’t read the letter in English. So, a lot of my job was reading the letter to an interpreter on the phone and explaining it to the patient … Or telephone appointments without times so we can’t organise a translator Participant 2

Doubts about the quality of interpretation and an imperative to use time efficiently resulted in some participants avoiding telephone interpretation. Instead, they improvised with non-verbal cues, gesticulations and mobile phone apps. This approach is evocative of ‘tinkering’, described by Mol,^38^ as a way of adjusting towards situationally-determined improved outcomes:

I think I was very much like, I don’t want to use this unless I really have to … I tried to communicate like, with facial expressions and hand gestures Participant 5

we just didn’t have the time [to use telephone interpretation] … we started to use the phone app … I’m sure that it affected care. Participant 2

Recognising the importance of good communication for safe care, participants were driven to search for workarounds in the face of limited time, resources and poor-quality interpretation services. Many ‘tinkered’ to moderate and simplify communication:

it’s about how to simplify the language and talk more slowly and talk in shorter sentences because I was going through a translator Participant 9

being resourceful, that is, drawing pictures on paper, using body language or hand gestures as signals and things like that. It is not always easy because of COVID, and you are wearing a face mask … sometimes I use Google Translate or ‘Siri’ will speak. Participant 10

Although some framed this innovation in a positive light, others suggested how this might compromise care:

[some people] they would translate for their friends, which again is an issue with confidentiality and safety at times. I’m not sure if I would want to disclose some things to my friends. That was a last resort … I guess things could have been missed if people didn’t want to disclose some information, but that’s how we got around it Participant 2

Again, the insufficient provision of interpretation was not articulated as discrimination.

## Discussion

This qualitative analysis illustrates how staff understood the disadvantage experienced by those with LEP differently from that based on migration status or ethnicity. When discrimination based on migration status was identified, some staff saw calling it out as part of their role and successfully facilitated change. In contrast, the impact of inadequate interpretation on providing safe and trauma-informed care was not identified as discriminatory, despite all of those interviewed recognising the risks. Consequently, it was not challenged in the same way. Instead, when faced with a lack of available face-to-face interpreters and poor-quality difficult-to-access telephone interpretation, staff ‘tinkered’, using workarounds such as translation apps, gesticulating or drawings.

The concept of ‘tinkering’ has been used to explore care practices in various resource-poor contexts.^38 39^ Recently, ethnographic work has demonstrated the resourcefulness of healthcare staff and the necessity of creative innovations in low-income settings: ‘[i]f the system doesn’t work, you have to make it work for you, for the sake of the patient’.^40^ However, tinkering has limits—Reider describes how it can lead to physician disenfranchisement and departure.^41^ By framing these practices through the lens of language discrimination, we add to this critical perspective on the practice of tinkering. When staff feel workarounds are their only option, it seems that ‘tinkering’ can become usual practice, even in resource-rich countries where healthcare faces ideologically driven cuts. Inequalities are thus reproduced, normalised and embedded within organisational structures and the ability to change discriminatory systems is diminished.

US-based research links the concept of language discrimination and patients’ poor experiences of healthcare access.^42 43^ However, we appear to be the first to apply the sociological concept of language discrimination to the UK healthcare setting, use it to critically analyse healthcare providers’ narratives of care provision, and argue for its more widespread application as a means to address inequalities. In doing so, we make an important theoretical contribution. Empirically, language discrimination is evident throughout this data: differential treatment on the grounds of LEP perpetuates inequalities in care and maintains the normative hierarchies of power and privilege, where people who speak English receive better care. We caution against the assumption that language is a modifiable characteristic and therefore not challenged as readily as other forms of discrimination, such as racism. This speculation overlooks the well-described structural, psychological, social and educational barriers to learning new languages.^44^

Antiracist scholars assert that naming discrimination and examining how it operates are the first steps in confronting inequitable health systems.^31^ We uphold that promoting the application of the concept of language discrimination is the first step in addressing inequitable care for those with LEP. Naming inadequate interpretation provision in healthcare as a form of discrimination works to overcome institutional and social inertia, empowering staff and giving them vocabulary to challenge health inequalities experienced by people with LEP. In the contemporary UK context, the gap in interpretation provision can be understood as part of a broader discriminatory approach to migrants. Technical solutions to improve care are needed, with provision of culturally sensitive interpreters as the bare minimum. Moreover, language discrimination is only one facet of the hostile environment facing migrants in the UK.^22^ Since undertaking this research, living conditions for people seeking asylum have been further eroded, with plans for offshore processing and accommodation on sea vessels.

Illuminating the issues from a practitioner perspective, this paper contributes to the literature on the underuse of interpreters in healthcare.^14^ We emphasise the chasm between academic knowledge, assertions from policy-makers and the reality of conditions facing practitioners. A wealth of literature documents the harms of inadequate interpretation alongside healthcare policy proclaiming that those with LEP should not experience worse care.^16 17^ Yet when healthcare staff face high clinical workloads without access to user-friendly high-quality interpretation, using workarounds to get the job done becomes usual practice and inequity becomes institutionalised. It is important to note that in countries where payment is structured differently the extra time required for interpretation may also impact clinician income.

Our empirical findings illuminate why health outcomes are pitiful when provision of interpretation services is inadequate and shed light on the variable uptake of healthcare services across diverse communities. They should interest practitioners and researchers concerned with structural causes of health inequalities. Our analysis highlights that some social groups are underserved rather than ‘difficult to reach’. Of relevance for policy-makers is the lack of transparency around commissioning for interpretation provision in England and the marked variability in expenditure for comparable demographics.^45 46^ A radical overhaul of interpretation provision is crucial to address ethnic inequalities in health.

This paper focuses on language discrimination, a previously underexplored yet important component of ethnic health inequalities. The qualitative data is rich and extensive; however, the study does have limitations. The number of participants is modest due to the specialist nature of the service. We did not collect data about the languages spoken by healthcare staff or how patients’ English language proficiency was assessed, both are areas which warrant further investigation. There may be social desirability bias of participants’ self-representation to the interviewer, who was a clinician and researcher. These biases could arguably make the findings more salient. The staff at this specialist service often went above and beyond usual care and it is likely that the normalisation and institutionalisation identified here would be readily replicated elsewhere by less committed individuals. Research exploring how these findings translate to other settings would be valuable.

## Conclusion

This research provides empirical evidence of how the inadequate provision of interpreters, shown here in the context of the pandemic, appears to force the hand of healthcare staff to use shortcuts. This risks compromising the safe care of people with LEP. Both structural and operational changes are needed to improve healthcare provision for migrants. Inadequate interpretation provision should be understood as part of a complex system of discrimination facing people seeking asylum which requires a radical overhaul. More specifically, policy-makers must rethink their approach to interpretation provision including training of practitioners to work with interpreters, integrating systems to reduce factors that prevent their use, and prioritising quality over cost to ensure safety.

Endorsement of the concept of language discrimination in healthcare would provide a useful framework for clinicians to better identify and articulate unfair treatment on the grounds of LEP. Borrowing from antiracist scholarship, we argue that this is the first step in shifting the culture around the ubiquitous but hidden acceptance of a poor standard of care for LEP patients. Staff were tenacious and challenged practices where more overt examples of discrimination were experienced and could be named. Future research should examine healthcare workers’ perception of the concept of language discrimination and the utility and limitations of the concept in addressing health inequalities.

## Supplementary Material

Reviewer comments

Author's manuscript

## Data Availability

Data are available on reasonable request. No data are available. Data contains sensitive information. Please contact lead author to discuss access.
