# Supplementary material for: ‘That’s how we got around it’: a qualitative exploration of healthcare professionals’ experiences of care provision for asylum applicants’ with limited English proficiency in UK contingency accommodation
Source: BMJ Open. 2023 Nov 23;13(11):e074824. doi: 10.1136/bmjopen-2023-074824 (PMC10668227; doi:10.1136/bmjopen-2023-074824)
Supplement: Author's manuscript [file bmjopen-2023-074824.draft_revisions.pdf]

BMJ Open is committed to open peer review. As part of this commitment we make the peer review history of every article we publish publicly available.

When an article is published we post the peer reviewers' comments and the authors' responses online. We also post the versions of the paper that were used during peer review. These are the versions that the peer review comments apply to.

The versions of the paper that follow are the versions that were submitted during the peer review process. They are not the versions of record or the final published versions. They should not be cited or distributed as the published version of this manuscript.

BMJ Open is an open access journal and the full, final, typeset and author-corrected version of record of the manuscript is available on our site with no access controls, subscription charges or pay-per-view fees (http://bmjopen.bmj.com).

If you have any questions on BMJ Open's open peer review process please email info.bmjopen@bmj.com

BMJ Open

‘That’s how we got around it’: UK healthcare professionals’ work with asylum applicants with limited English proficiency during the pandemic

Journal:	*BMJ Open*
Manuscript ID	bmjopen-2023-074824
Article Type:	Original research
Date Submitted by the Author:	18-Apr-2023

Complete List of Authors:	Tomkow, Louise; The University of Manchester,<br>Prager, Gabrielle; Johns Hopkins University<br>Drinkwater, Jessica; The University of Manchester, Centre for Primary Care and Health Services Research<br>Morris, Rebecca L.; The University of Manchester, Centre for Primary Care<br>Farrington, Rebecca; University of Manchester Faculty of Biology, Medicine and Health, Division of Medical Education

Keywords:	Health Services Accessibility, Primary Health Care, Health Equity, Quality in health care < HEALTH SERVICES ADMINISTRATION & MANAGEMENT, Organisation of health services < HEALTH SERVICES ADMINISTRATION & MANAGEMENT

SCHOLARONE™
Manuscripts

I, the Submitting Author has the right to grant and does grant on behalf of all authors of the Work (as defined in the below author licence), an exclusive licence and/or a non-exclusive licence for contributions from authors who are: i) UK Crown employees; ii) where BMJ has agreed a CC-BY licence shall apply, and/or iii) in accordance with the terms applicable for US Federal Government officers or employees acting as part of their official duties; on a worldwide, perpetual, irrevocable, royalty-free basis to BMJ Publishing Group Ltd ("BMJ") its licensees and where the relevant Journal is co-owned by BMJ to the co-owners of the Journal, to publish the Work in this journal and any other BMJ products and to exploit all rights, as set out in our licence.

The Submitting Author accepts and understands that any supply made under these terms is made by BMJ to the Submitting Author unless you are acting as an employee on behalf of your employer or a postgraduate student of an affiliated institution which is paying any applicable article publishing charge ("APC") for Open Access articles. Where the Submitting Author wishes to make the Work available on an Open Access basis (and intends to pay the relevant APC), the terms of reuse of such Open Access shall be governed by a Creative Commons licence – details of these licences and which Creative Commons licence will apply to this Work are set out in our licence referred to above.

Other than as permitted in any relevant BMJ Author's Self Archiving Policies, I confirm this Work has not been accepted for publication elsewhere, is not being considered for publication elsewhere and does not duplicate material already published. I confirm all authors consent to publication of this Work and authorise the granting of this licence.

For peer review only

'That's how we got around it': UK healthcare professionals' work with asylum applicants with limited English proficiency during the pandemic

Abstract

Objectives

The inadequate provision of language interpretation for people with Limited English Proficiency (LEP) is a determinant of poor health, yet interpreters are underused. This research explores the experiences of NHS staff providing primary care for people with LEP: people seeking asylum, housed in contingency accommodation during COVID-19 - a group known to face multiple healthcare access barriers. Language discrimination is used as a theoretical framework and the potential utility of this concept is explored as a way of understanding and addressing inequities in care.

Design

Qualitative research using semi-structured interviews and inductive thematic analysis.

Setting

Healthcare staff from NHS primary care service for people seeking asylum based in contingency accommodation during COVID-19.

Participants

Ten staff, including doctors, nurses, mental health practitioners, health care assistants and students participated in semi-structured online interviews. Some staff were redeployed to this work due to the pandemic.

Results

Patients' LEP was significant for all interviewees. Inadequate provision of interpretation services impacted the staff's ability to provide care and compromised patient safety. Other forms of discrimination, such as that based on migration status, were recognised and challenged by staff. However, inequity based on language was not articulated as discrimination. Instead, insufficient and substandard interpretation was accepted as the status quo and workarounds used, such as gesticulating or phone apps. The theoretical

For peer review only - http://bmjopen.bmj.com/site/about/guidelines.xhtml

lens of language discrimination shows how this propagates existing social hierarchies and further disadvantages those with LEP.

Conclusions

This research provides empirical evidence of how the inadequate provision of interpreters forces the hand of healthcare staff to use shortcuts. It risks compromising care for people with LEP. Policymakers must rethink their current approach to interpretation provision which prioritises costs over quality. We assert that the concept of language discrimination is a valuable framework for clinicians to better identify and articulate unfair treatment on the grounds of LEP.

Strengths and limitations

- This paper focuses on language discrimination, a previously under-explored yet important component of ethnic health inequalities
- The qualitative data is rich and in depth
- The paper makes novel empirical and theoretical contributions
- The limited sample size is due to the specialist nature of the service
- Research exploring how these findings operate in other settings would be valuable.

For peer review only - http://bmjopen.bmj.com/site/about/guidelines.xhtml

Introduction

Good-quality communication between healthcare providers and patients is crucial for safe person-centred care.[1] Although we know the harmful effects of poor communication on care outcomes, there is scarce research focusing on clinicians' experiences and negotiation of language barriers. In English-speaking countries worldwide, including Australia, the US and the UK, patients with Limited English Proficiency (LEP) experience barriers accessing care;[2,3] more medical errors;[4,5] lower satisfaction;[6] and significant misunderstanding of health information.[7]

The benefits of providing high-quality interpretation include: improved clinical care;[8,9] reduced inequalities in healthcare access;[10] and cost savings through decreased hospital readmissions and length of stay.[11,12] Despite this, interpretation services remain underused.[13] This is justified by time constraints, lack of availability of interpreters, and dissatisfaction with the quality of interpretation.[13] Worryingly, a study exploring doctors' reasoning suggested that a clinician-centred approach to communication might be contributory - for some doctors, communication was merely seen as a way of collecting information.[14]

Addressing population health inequalities is touted as a priority for the UK's NHS and is foregrounded in pandemic recovery policy.[15,16] NHS England outlines key principles for the provision of high-quality interpretation and translation services.[17] However, a recent UK review identified that longstanding ethnic inequalities in access, experience and healthcare outcomes are due, in part, to a continued lack of appropriate interpretation services.[18]

Research context

This paper examines the experiences of NHS staff during the COVID-19 pandemic working in UK asylum contingency accommodation and communicating with people with LEP. People seeking asylum face multiple obstacles to accessing healthcare and frequently have diverse and additional needs to settled populations.[2] Inadequate interpretation is a significant barrier.[19] In addition, migrants to the UK have faced increasingly restrictive welfare rights over the past decade, including some restrictions to entitlements for free NHS care. There is confusion amongst NHS care providers about charging migrants and people seeking asylum often lack awareness of how to navigate the NHS.[20] Even if they do negotiate

For peer review only

these challenges, the time-limited structure of NHS consultations can be inadequate to address their complex needs.[21]

The COVID-19 pandemic augmented the difficulties, both in terms of living conditions and healthcare access. The Home Office's use of contingency accommodation, such as hotels and army barracks, increased considerably during the pandemic.[22] Healthcare access for people seeking asylum housed this way was documented as inadequate pre-pandemic; evidence suggests this has since deteriorated significantly and concerns continue to be raised regarding unsafe conditions.[23] During the pandemic, many NHS services adopted blanket remote working, worsening existing barriers to access and issues with communication.[24] In recognition, some primary care services established extra services focusing on the health needs of those in contingency accommodation.[25] This paper examines NHS staff experiences at one such primary care service, rapidly implemented in Spring 2020, based on-site for over 200 people seeking asylum, and providing face-to-face care throughout the pandemic. Many of the staff were redeployed as part of the COVID-19 response.

Research aims

This research aims to investigate the experiences of NHS staff during COVID-19, examining how staff understood, experienced and negotiated care provision for people seeking asylum with LEP. As a secondary aim, we scrutinise how staff understood discrimination towards patients and explore the potential utility of the concept of language discrimination as a way of comprehending inequities in access to care. The considerable impact of inadequate interpretation provision on healthcare experiences and health outcomes endorses this as an area in urgent need of action.

Theoretical framework

We use the concept of language discrimination as a theoretical framework.[26] For the purposes of this paper, we understand this concept as articulated by Lippi Green: a sociological theoretical lens to observe the extent to which discrimination on the grounds of LEP serves to perpetuate inequitable treatment and reinforces normative power hierarchies. We do so from an anti-racist position that embraces naming discrimination, then asking 'how is it operating here?' as the first steps to addressing it.[27]

For peer review only - http://bmjopen.bmj.com/site/about/guidelines.xhtml

Lippi-Green's work examines how discrimination on the grounds of accent or LEP serves to perpetuate normative power hierarchies.[26] That is, how those who speak English as a first language consistently benefit over those with LEP. US studies have shown how language discrimination causes individuals to feel overlooked and inferior,[28] and is associated with adverse health outcomes,[29] but there is little exploration of how language discrimination operates beyond the USA. The scarcity of work describing language discrimination in UK healthcare is perhaps related to opacity over the legal protection offered to those with LEP, as it is not considered a 'protected characteristic' under the Equality Act 2010. However, both socially and legally, language is inextricably intertwined with nationality and race which are protected characteristics.[29,30,31,32] As a result, NHS England's interpretation commissioning guidance acknowledges discrimination on the basis of national origin through a lack of language assistance for LEP persons.[17] Consequently, we must view it as an essential strategic consideration in reducing health inequalities.

Methods

Study design

Twenty-seven healthcare professionals currently or previously working with the asylum seeker primary care service were invited via email to participate in an online interview about their experiences. In 2021, ten of these healthcare professionals participated in remote semi-structured qualitative interviews, lasting between 45 minutes and 2.5 hours. Participants included doctors, nurses, mental health practitioners, health care assistants and students, some of whom had been redeployed to primary care in the pandemic. Interviews were conducted using Zoom video conferencing software by author GP, a clinical academic with no prior relationship to the invited healthcare professionals. Interviews began with verbal consent and participants shared a brief explanation of their professional background and role. Participants were told that the research team were interested in migrant health and their experiences working at the service during COVID-19. Interviews were conversational, with open questioning using an interview guide on the practical and ethical issues of care provision in contingency accommodation. They allowed opportunities for participants to discuss issues and experiences important to them. The interview guide was developed in collaboration with health professionals experienced in migrant health work. Audio of the interviews was recorded, notes taken by the interviewer, and recordings transcribed verbatim by GP. No payment was offered for participation. Data was anonymised, with place names changed and pseudonyms to protect participants. Demographic details for individual participants are not shared due

For peer review only - http://bmjopen.bmj.com/site/about/guidelines.xhtml

to the small sample size, the specialist nature of the service, and to maintain confidentiality. Two site visits were undertaken by the researchers (LT and GP) to familiarise with the environment.

Data analysis

Using inductive thematic analysis, both LT and GP coded data using NVivo 12 to increase robustness.[33] Dominant themes were derived from the transcripts before data was coded and extracted. First phase analysis focussed on barriers and mitigations of providing care in contingency accommodation and is explored elsewhere (*in press*). Language and discrimination appeared as key themes and prompted additional examination. Secondary thematic analysis was therefore applied to the data using Lippi-Green's language discrimination as a theoretical framework (by GP, LT, JD, RM and RF). This occurred after data collection as specific questions about language and discrimination were not in the interview topic guide. The University of xxxxx Research Ethics Committee gave approval (NHS001845).

Research team and reflexivity

The research team are clinical academics with an interest in migrant health. LT (PI) and RF designed and led the research. LT developed study materials and ethics application. LT supervised GP in recruitment and interviews. All authors (LT, GP, JD, RM and RF) contributed to the analysis. Our interest in migrant health risks the imposition of our own beliefs on the research. LT and RF work in migrant health advocacy which facilitated access to the participants but could also shape both the data interpretation and the ideas in this paper. The clinical identity of the interviewer (GP) may have promoted a social desirability bias from participants. In mitigation, the interview topic guide was developed with several clinicians.

Results

All participants stressed the importance of patients' LEP and the inadequate provision of interpretation services on care provision. Many commented on how patients were disadvantaged on the grounds of language and how this compromised patient safety. However, unlike other forms of discrimination such as racism, the disadvantage faced by people with LEP was rarely described in terms of discrimination. Instead, this disadvantage was normalised: inadequate and substandard interpretation was accepted as the status quo and workarounds were used.

For peer review only - http://bmjopen.bmj.com/site/about/guidelines.xhtml

To illustrate, we present three emergent themes from the data: (i) recognising and resisting discrimination; (ii) the importance of language in care provision and; (iii) normalising inadequate interpretation.

(i) Recognising and resisting discrimination

Healthcare staff readily recognised that people seeking asylum encountered discrimination on the grounds of migration status, nationality, and race:

> *'They will face a lot of discrimination in the quality [of care] … It's been a rude awakening … there's a lot of prejudice and stereotypes of those seeking asylum.'* Participant 7

Many noticed discrimination towards their patients from healthcare staff in other settings such as the pharmacy, hospital, and social care. Staff reported inappropriate questioning of patients' entitlement to NHS treatments and social care by service providers, resulting in some being denied care which they had the right to receive. Several portrayed how the local dental surgery had refused to see residents from contingency accommodation and this was labelled as discriminatory by participants. One participant used these allegations of discrimination as a way of challenging the dental surgery staff and advocating for all the residents:

> *'I said, "Can I just get this straight? So, you're telling me that one individual was abusive towards your staff, and because of that, you're going to be discriminating against 250 other individuals because you're deciding that everybody who is seeking asylum must be the same and is going to behave like this one person. Is that what you're saying?" … "No, no, no. That's not what we're saying." "Okay, good. Because that would have been discrimination, wouldn't it." These are the conversations that we have to have all of the time.'* Participant 1

This illustrates how recognising inequity as discrimination - and articulating it as such - allows individuals to challenge and resist inequitable treatment. Importantly, it also demonstrates that some healthcare staff recognise calling out discrimination as part of their role.

For peer review only - http://bmjopen.bmj.com/site/about/guidelines.xhtml

(ii) The importance of language in care provision

When asked about wider experiences working in the contingency accommodation, all participants spontaneously stressed the importance of LEP and contrasted it with their previous NHS work. They acknowledged how LEP compounded the multiple complex needs of their asylum-seeking patients and the importance of language interpretation services to reduce inequity:

> *'I mean, in my view, [translation services] should be a bare minimum for any service delivering health care in the UK ... Because otherwise, you create instant inequalities. Particularly within this very, very vulnerable group of people. Why that isn't a national standard? I don't know.'* Participant 1

> *'It's quite chaotic … The difference, obviously the patient group have a lot of needs that need to be addressed and to be managed, especially the fact that English is not their first language.'* Participant 7

Participants frequently associated inadequate interpretation provision with inequity in care, even highlighting safety concerns such as COVID-19 transmission risk, illustrating the wider public health implications of the derisory provision of information to patients with LEP:

> *'In the really early days when they were not providing information about COVID in their own languages, a lot of them didn't know what COVID was'.* Participant 10

Participant 10 also draws attention to the risks posed by medication errors:

> *'The patients here didn't speak English. They can't read road signs, they don't know where the GP practice is, or the pharmacy is. And when they get to the pharmacy, they can't communicate with the lady behind the desk, so it was really difficult in that sense, and then they would get the medication and be like, "I can't read the label. How do I take this?" And then there are safety aspects to that in that they are not ingesting their medication in the right way and things like that, so that was difficult, so things that are normally easy are really, really hard.'* Participant 10

For peer review only

This excerpt illustrates the multiple structural barriers faced by patients with LEP trying to navigate health systems, access care and to self-manage. It demonstrates the need for improvements in interpretation services beyond medical consultations. LEP instigates barriers to accessing safe care and achieving good health that are not encountered by English speakers. This fits with the theoretical framework of language discrimination as a means of highlighting the propagation of existing social hierarchies. Although participants recognised clear inequities they did not directly articulate them as being based in discriminatory practice.

For peer review only

(iii) Normalising inadequate interpretation provision

All participants faced challenges accessing adequate interpretation services. Many described the resulting suboptimal care and inequities. As part of the pandemic response, community volunteers initially provided face-to-face interpretation at the contingency accommodation for medical consultations. This in-person service was described by Participant 2 as '*so useful … made life so much easier … made such a difference*'. However, this was not possible beyond the first few months as their own voluntary organisations reopened. The clinical team were left using telephone interpretation services, universally reported as problematic and often labelled a *'waste'* of time:

> *'The waits for [the telephone interpretation service] were quite extensive. 30 minutes, 45 minutes, which had a huge impact on clinical time. And you would often get through and then not be able to get through to the patient. We were wasting a huge number of clinical hours at that point in just trying to get hold of patients and speak to them.'* Participant 1

In addition, the poor mobile phone signal in the accommodation caused frequent and lengthy interruptions. Unfortunately, even when staff were able to connect, the quality of interpretation was variable and often substandard:

> *'Someone would talk at length sometimes and the interpreter would summarise it in a few sentences, so I don't know if I was actually getting the full picture … sometimes because [patients] had partial English they would sometimes say 'I'm not saying that'.'* Participant 9

For peer review only - http://bmjopen.bmj.com/site/about/guidelines.xhtml

Inadequate interpretation services in secondary care also impacted the workload of the primary care participants. Here they took time in an already pressured environment to fill the void left by inadequate language provision:

> *'The doctor sends the referrals, and then the patient gets a letter, but they can't read the letter in English. So, a lot of my job was reading the letter to an interpreter on the phone and explaining it to the patient … Or telephone appointments without times so we can't organise a translator'* Participant 2

Doubts about the quality of interpretation and an imperative to use time efficiently to help as many people as possible resulted in some participants avoiding the use of telephone interpretation. Instead, they improvised with non-verbal cues and mobile phone apps:

> *'I think I was very much like, I don't want to use this unless I really have to … I tried to communicate like, with facial expressions and hand gestures'* Participant 5

> *'we just didn't have the time [to use telephone interpretation] … we started to use the phone app … I'm sure that it affected care.'* Participant 2

Many used 'workarounds' to moderate and simplify communication. An awareness of compromises in standards of care is clearly illustrated in the narrative:

> *'[some people] they would translate for their friends, which again is an issue with confidentiality and safety at times. I'm not sure if I would want to disclose some things to my friends. That was a last resort … I guess things could have been missed if people didn't want to disclose some information, but that's how we got around it'* Participant 2

> *'it's about how to simplify the language and talk more slowly and talk in shorter sentences because I was going through a translator'* Participant 9

For peer review only - http://bmjopen.bmj.com/site/about/guidelines.xhtml

'being resourceful, i.e. drawing pictures on paper, using body language or hand gestures as signals and things like that. It is not always easy because of COVID, and you are wearing a face mask … sometimes I use google translate or 'Siri' will speak.' Participant 10

These accounts indicate the participants' resigned acceptance of sub-standard inequitable communication for people with LEP in order just to get the basics of 'the job' done. Recognising the importance of good communication for excellent care, they were driven to search for workarounds in the face of limited time, resources and poor-quality interpretation services. These workarounds became usual practice as the narratives reveal participants felt these became their only options. Their inability to challenge poor quality communication through changing the systems, and its consequent inequity, is implicit. Again, the insufficient provision of interpretation was not articulated as discrimination.

For peer review only

For peer review only - http://bmjopen.bmj.com/site/about/guidelines.xhtml

Discussion

The qualitative data in this paper illustrates how staff understood the disadvantage experienced by those with LEP differently from that based on migration status or ethnicity. When discrimination based on migration status was recognised, some staff saw calling it out as part of their role and successfully facilitated change. In contrast, they did not articulate the impact of inadequate interpretation on safe care as discriminatory despite all of the staff interviewed recognising how the care provided to patients with LEP was inferior to those speaking English. Consequently, it was not challenged in the same way. Faced with a lack of available face-to-face interpreters and poor-quality difficult-to-access telephone interpretation, staff instead resorted to workarounds such as using translation apps, gesticulating or using drawings. Thus despite their best efforts, inequitable and suboptimal care for those with LEP was unintentionally normalised.

We appear to be the first to apply the sociological concept of language discrimination to the UK healthcare setting. In doing so, this paper makes an important theoretical contribution. Language discrimination is evident throughout the data: differential treatment on the grounds of LEP perpetuates inequalities in care and maintains the normative power hierarchies where people who speak English receive better care. We caution against the assumption that language discrimination is not challenged as readily as other forms of discrimination, such as racism, because language may be considered a modifiable social characteristic. This speculation overlooks the well-described structural, psychological, social and educational barriers to learning a new language.[34]

Anti-racist scholars assert that naming discrimination and examining how it operates are the first steps in confronting inequitable health systems.[27] We, therefore, uphold that promoting the application of the concept of language discrimination is the first step in addressing inequitable care for those with LEP. Naming inadequate interpretation provision in healthcare as a form of discrimination works to overcome institutional and social inertia, empowering staff and giving them the vocabulary to challenge health inequalities experienced by people with LEP.

Illuminating the issues from a practitioner perspective, this paper makes an important contribution to the literature on the underuse of interpreters in healthcare.[14] We emphasise the chasm between academic knowledge, assertions from policymakers, and the reality of conditions practitioners face. A wealth of

For peer review only - http://bmjopen.bmj.com/site/about/guidelines.xhtml

literature documents the harms of inadequate interpretation and healthcare policy proclaims that those with LEP should not face worse care.[16,17] Yet when healthcare staff face high clinical workloads without access to user-friendly high-quality interpretation, using workarounds to get the job done becomes usual practice and inequity becomes institutionalised.

Our empirical findings illuminate why health outcomes are pitiful when provision of interpretation services is inadequate and shed light on the variable uptake of healthcare services across different communities. They should interest practitioners and researchers concerned with the structural causes of health inequalities. Our analysis highlights that some social groups are underserved and not 'difficult to reach'. Of particular relevance for policymakers is the lack of transparency around services commissioning for interpretation provision in England and the marked variability in expenditure for comparable demographics (35). A radical overhaul of interpretation provision is crucial to address ethnic inequalities in health.

This paper focuses on language discrimination, a previously under-explored yet important component of ethnic health inequalities. The qualitative data is rich and extensive; however, the study does have limitations. The two most significant are the limited sample size due to the specialist nature of the service; and the social desirability bias of participants' self-representation to the interviewer, who was a clinician and researcher. These biases could arguably make the findings more salient. The staff at this specialist service often went above and beyond usual care and it is likely that the normalisation and institutionalisation identified here would readily be replicated elsewhere by less committed individuals. Research exploring how these findings translate to other settings would be valuable.

Conclusion

This research provides empirical evidence of how the inadequate provision of interpreters, shown here in the context of the pandemic, appears to force the hand of healthcare staff to use shortcuts and risks compromising the care of people with LEP. Policymakers must rethink the current approach to interpretation provision which prioritises costs over quality. We advocate that increasing the uptake of the concept of language discrimination in healthcare would provide a useful framework on which clinicians can better identify and articulate unfair treatment on the grounds of LEP. Borrowing from anti-racist scholarship, we argue that this is the first step in shifting the culture around the standard of care

For peer review only - http://bmjopen.bmj.com/site/about/guidelines.xhtml

for LEP patients. Staff were tenacious and challenged discriminatory practice where more overt examples of discrimination were experienced and named. Future research should examine healthcare workers' perception of the concept of language discrimination, and the utility and limitations of the concept in addressing health inequalities.

References

1. Ratna H. The importance of effective communication in healthcare practice. Harvard Public Health Review. 2019 Oct 1;23:1-6.
2. Kang C, Tomkow L, Farrington R. Access to primary health care for asylum seekers and refugees: a qualitative study of service user experiences in the UK. British Journal of General Practice. 2019 Aug 1;69(685):e537-45.
3. White J, Plompen T, Osadnik C, Tao L, Micallef E, Haines T. The experience of interpreter access and language discordant clinical encounters in Australian health care: a mixed methods exploration. International journal for equity in health. 2018 Dec;17(1):1-0.
4. Divi C, Koss RG, Schmaltz SP, Loeb JM. Language proficiency and adverse events in US hospitals: a pilot study. International journal for quality in health care. 2007 Apr 1;19(2):60-7.
5. Flores G, Laws MB, Mayo SJ, Zuckerman B, Abreu M, Medina L, Hardt EJ. Errors in medical interpretation and their potential clinical consequences in pediatric encounters. Pediatrics. 2003 Jan;111(1):6-14.
6. Baker DW, Hayes R, Fortier JP. Interpreter use and satisfaction with interpersonal aspects of care for Spanish-speaking patients. Medical care. 1998 Oct 1:1461-70.
7. Wilson E, Chen AH, Grumbach K, Wang F, Fernandez A. Effects of limited English proficiency and physician language on health care comprehension. Journal of general internal medicine. 2005 Sep;20(9):800-6.
8. Karliner LS, Jacobs EA, Chen AH, Mutha S. Do professional interpreters improve clinical care for patients with limited English proficiency? A systematic review of the literature. Health services research. 2007 Apr;42(2):727-54.
9. Lee JS, Pérez-Stable EJ, Gregorich SE, Crawford MH, Green A, Livaudais-Toman J, Karliner LS. Increased access to professional interpreters in the hospital improves informed consent for patients with limited English proficiency. Journal of general internal medicine. 2017 Aug;32(8):863-70.

For peer review only

10. Jacobs EA, Lauderdale DS, Meltzer D, Shorey JM, Levinson W, Thisted RA. Impact of interpreter services on delivery of health care to limited-English-proficient patients. Journal of general internal medicine. 2001 Jul;16(7):468-74.

11. Karliner LS, Pérez-Stable EJ, Gregorich SE. Convenient access to professional interpreters in the hospital decreases readmission rates and estimated hospital expenditures for patients with limited English proficiency. Medical care. 2017 Mar;55(3):199.

12. John-Baptiste A, Naglie G, Tomlinson G, Alibhai SM, Etchells E, Cheung A, Kapral M, Gold WL, Abrams H, Bacchus M, Krahn M. The effect of English language proficiency on length of stay and in-hospital mortality. Journal of general internal medicine. 2004 Mar;19(3):221-8.

13. Kale E, Syed HR. Language barriers and the use of interpreters in the public health services. A questionnaire-based survey. Patient education and counseling. 2010 Nov 1;81(2):187-91.

14. Diamond LC, Schenker Y, Curry L, Bradley EH, Fernandez A. Getting by: underuse of interpreters by resident physicians. Journal of general internal medicine. 2009 Feb;24(2):256-62.

15. NHS Long term plan, NHE England. Available at: https://www.england.nhs.uk/long-term-plan/ Accessed 13.10.22

16. Ethnic Inequalities in Healthcare: A Rapid Evidence Review. NHS race and health observatory. Available at: https://www.nhsrho.org/wp-content/uploads/2022/02/RHO-Rapid-Review-Final-Report_Summary_v.4.pdf Accessed 13.10.22

17. Guidance for commissioners interpreting and translation services in primary care, NHS England. Available at: https://www.england.nhs.uk/wp-content/uploads/2018/09/guidance-for-commissioners-interpreting-and-translation-services-in-primary-care.pdf Accessed 13.10.22

18. Patel KC, Hanif W. Ethnic health inequalities in the NHS. BMJ. 2022 Mar 9;376.

19. Patel P, Bernays S, Dolan H, Muscat DM, Trevena L. Communication experiences in primary healthcare with refugees and asylum seekers: a literature review and narrative synthesis. International journal of environmental research and public health. 2021 Feb;18(4):1469.

20. Tomkow LJ, Kang CP, Farrington RL, Wiggans RE, Wilson RJ, Pushkar P, Tickell-Painter MC, Lee AR, Whitehouse ER, Mahmood NG, Lawton KM. Healthcare access for asylum seekers and refugees in England: a mixed methods study exploring service users' and health care professionals' awareness. European Journal of Public Health. 2020 Jun 1;30(3):527-32.

21. Arnold FW. How can we help asylum seekers get better healthcare?. bmj. 2022 Feb 7;376.

For peer review only - http://bmjopen.bmj.com/site/about/guidelines.xhtml

22. The Home Office. The use of temporary hotels to house asylum seekers during Covid-19. August 2020. Available at: https://homeofficemedia.blog.gov.uk/2020/08/08/the-use-of-temporary-hotels-to-house-asylum-seekers-during-covid-19/ Accessed August 2022
23. Jones L, Phillimore J, Fu L, Hourani J, Lessard-Phillips L, Tatem B. Doctors of the World Report. *They just left me Asylum seekers, health, and access to healthcare in initial and contingency accommodation. 2022.* Report. Available at: https://www.doctorsoftheworld.org.uk/wp-content/uploads/2022/04/DOTW-Access-to-healthcare-in-initial-and-contingency-accommodation-report-April-2022.pdf. Accessed March 2023
24. Written evidence submitted by Doctors of the World (DOTW) (COR0203) [Internet]. [cited 2022 Mar 28]. Available from: https://bjgp.org/content/69/685/e537#ref-6
25. Farrant O, Eisen S, Van Tulleken C, Ward A, Longley N. Why asylum seekers deserve better healthcare, and how we can give it to them. bmj. 2022 Jan 6;376.
26. Lippi-Green R. English with an accent: Language, ideology, and discrimination in the United States. Routledge; 2012 Mar 15.
27. Jones CP. Confronting institutionalized racism. Phylon (1960-). 2002 Jan 1:7-22.
28. Lee JJ, Rice C. Welcome to America? International student perceptions of discrimination. Higher education. 2007 Mar;53(3):381-409.
29. Yoo HC, Gee GC, Takeuchi D. Discrimination and health among Asian American immigrants: Disentangling racial from language discrimination. Social science & medicine. 2009 Feb 1;68(4):726-32.
30. Language requirements can be a legal minefield. Employment Law. Available at:
31. https://www.peoplemanagement.co.uk/article/1745542/language-requirements-legal-minefield Accessed 13.10.22
32. Core20PLUS5 – An approach to reducing health inequalities. Available at: https://www.england.nhs.uk/about/equality/equality-hub/national-healthcare-inequalities-improvement-programme/core20plus5/#:~:text=Core20PLUS5%20is%20a%20national%20NHS,clinical%20areas%20requiring%20accelerated%20improvement Accessed 9.11.22
33. Thomas DR. A general inductive approach for analyzing qualitative evaluation data. American journal of evaluation. 2006 Jun;27(2):237-46.

For peer review only - http://bmjopen.bmj.com/site/about/guidelines.xhtml

34. Morrice L, Tip LK, Collyer M, Brown R. ‘You can’t have a good integration when you don’t have a good communication’: English-language learning among resettled refugees in England. Journal of Refugee Studies. 2021 Jun 26;34(1):681-99.
35. Lehane D, Oliver P, Mitchell C, Burton C. Interpreter costs across clinical commissioning groups in England 2017–2018: a cross sectional survey using ‘freedom of information’requests. British Journal of General Practice. 2020 Jun 1;70(suppl 1).

For peer review only

COMPETING INTERESTS

None

FUNDING

No specific funding to report for this work. GP is an Academic Clinical Fellow and LT and JD are Clinical Lecturers funded by Health Education England (HEE) and NIHR. RM’s input was supported by the National Institute for health and care Research (NIHR) Greater Manchester Patient Safety Translational Researcg Centre (award number PSTRC-2016-003). The views expressed in this publication are those of the author(s) and not necessarily those of the NIHR, NHS or the UK Department of Health and Social Care.

For peer review only - http://bmjopen.bmj.com/site/about/guidelines.xhtml

Standards for Reporting Qualitative Research (SRQR)*

http://www.equator-network.org/reporting-guidelines/srqr/

Title and abstract

	Page/line no(s).
**Title** - Concise description of the nature and topic of the study Identifying the study as qualitative or indicating the approach (e.g., ethnography, grounded theory) or data collection methods (e.g., interview, focus group) is recommended	Page 1 Line 1
**Abstract** - Summary of key elements of the study using the abstract format of the intended publication; typically includes background, purpose, methods, results, and conclusions	Page 1 lines 3 onwards

Introduction

**Problem formulation** - Description and significance of the problem/phenomenon studied; review of relevant theory and empirical work; problem statement	Pages 3-5
**Purpose or research question** - Purpose of the study and specific objectives or questions	Pages 3-5

Methods

**Qualitative approach and research paradigm** - Qualitative approach (e.g., ethnography, grounded theory, case study, phenomenology, narrative research) and guiding theory if appropriate; identifying the research paradigm (e.g., postpositivist, constructivist/ interpretivist) is also recommended; rationale**	Pages 5-6
**Researcher characteristics and reflexivity** - Researchers' characteristics that may influence the research, including personal attributes, qualifications/experience, relationship with participants, assumptions, and/or presuppositions; potential or actual interaction between researchers' characteristics and the research questions, approach, methods, results, and/or transferability	Page 6
**Context** - Setting/site and salient contextual factors; rationale**	Pages 3, 5-6
**Sampling strategy** - How and why research participants, documents, or events were selected; criteria for deciding when no further sampling was necessary (e.g., sampling saturation); rationale**	Page 5
**Ethical issues pertaining to human subjects** - Documentation of approval by an appropriate ethics review board and participant consent, or explanation for lack thereof; other confidentiality and data security issues	Page 6
**Data collection methods** - Types of data collected; details of data collection procedures including (as appropriate) start and stop dates of data collection and analysis, iterative process, triangulation of sources/methods, and modification of procedures in response to evolving study findings; rationale**	Pages 5-6

For peer review only

For peer review only - http://bmjopen.bmj.com/site/about/guidelines.xhtml

**Data collection instruments and technologies** - Description of instruments (e.g., interview guides, questionnaires) and devices (e.g., audio recorders) used for data collection; if/how the instrument(s) changed over the course of the study	Pages 5-6
**Units of study** - Number and relevant characteristics of participants, documents, or events included in the study; level of participation (could be reported in results)	Pages 5-6
**Data processing** - Methods for processing data prior to and during analysis, including transcription, data entry, data management and security, verification of data integrity, data coding, and anonymization/de-identification of excerpts	Pages 5-6
**Data analysis** - Process by which inferences, themes, etc., were identified and developed, including the researchers involved in data analysis; usually references a specific paradigm or approach; rationale**	Pages 5-6
**Techniques to enhance trustworthiness** - Techniques to enhance trustworthiness and credibility of data analysis (e.g., member checking, audit trail, triangulation); rationale**	Pages 5-6

Results/findings

**Synthesis and interpretation** - Main findings (e.g., interpretations, inferences, and themes); might include development of a theory or model, or integration with prior research or theory	Pages 6-11
**Links to empirical data** - Evidence (e.g., quotes, field notes, text excerpts, photographs) to substantiate analytic findings	Pages 6-11

Discussion

**Integration with prior work, implications, transferability, and contribution(s) to the field -** Short summary of main findings; explanation of how findings and conclusions connect to, support, elaborate on, or challenge conclusions of earlier scholarship; discussion of scope of application/generalizability; identification of unique contribution(s) to scholarship in a discipline or field	Pages 12-14
**Limitations** - Trustworthiness and limitations of findings	Page 13

Other

**Conflicts of interest** - Potential sources of influence or perceived influence on study conduct and conclusions; how these were managed	Page 17
**Funding** - Sources of funding and other support; role of funders in data collection, interpretation, and reporting	Page 17

*The authors created the SRQR by searching the literature to identify guidelines, reporting standards, and critical appraisal criteria for qualitative research; reviewing the reference lists of retrieved sources; and contacting experts to gain feedback. The SRQR aims to improve the transparency of all aspects of qualitative research by providing clear standards for reporting qualitative research.

For peer review only - http://bmjopen.bmj.com/site/about/guidelines.xhtml

**The rationale should briefly discuss the justification for choosing that theory, approach, method, or technique rather than other options available, the assumptions and limitations implicit in those choices, and how those choices influence study conclusions and transferability. As appropriate, the rationale for several items might be discussed together.

Reference:

O'Brien BC, Harris IB, Beckman TJ, Reed DA, Cook DA. **Standards for reporting qualitative research: a synthesis of recommendations.** *Academic Medicine*, Vol. 89, No. 9 / Sept 2014 DOI: 10.1097/ACM.0000000000000388

For peer review only

For peer review only - http://bmjopen.bmj.com/site/about/guidelines.xhtml

BMJ Open

'That's how we got around it': A qualitative exploration of healthcare professionals' experiences of care provision for asylum applicants' with Limited English Proficiency in UK contingency accommodation

Journal:	*BMJ Open*
Manuscript ID	bmjopen-2023-074824.R1
Article Type:	Original research
Date Submitted by the Author:	14-Sep-2023
Complete List of Authors:	Tomkow, Louise; The University of Manchester,<br>Prager, Gabrielle; Johns Hopkins University<br>Drinkwater, Jessica; The University of Manchester, Centre for Primary Care and Health Services Research<br>Morris, Rebecca L.; The University of Manchester, Centre for Primary Care<br>Farrington, Rebecca; University of Manchester Faculty of Biology, Medicine and Health, Division of Medical Education
**Primary Subject Heading**:	Communication
Secondary Subject Heading:	General practice / Family practice
Keywords:	Health Services Accessibility, Primary Health Care, Health Equity, Quality in health care < HEALTH SERVICES ADMINISTRATION & MANAGEMENT, Organisation of health services < HEALTH SERVICES ADMINISTRATION & MANAGEMENT

SCHOLARONE™
Manuscripts

I, the Submitting Author has the right to grant and does grant on behalf of all authors of the Work (as defined in the below author licence), an exclusive licence and/or a non-exclusive licence for contributions from authors who are: i) UK Crown employees; ii) where BMJ has agreed a CC-BY licence shall apply, and/or iii) in accordance with the terms applicable for US Federal Government officers or employees acting as part of their official duties; on a worldwide, perpetual, irrevocable, royalty-free basis to BMJ Publishing Group Ltd ("BMJ") its licensees and where the relevant Journal is co-owned by BMJ to the co-owners of the Journal, to publish the Work in this journal and any other BMJ products and to exploit all rights, as set out in our licence.

The Submitting Author accepts and understands that any supply made under these terms is made by BMJ to the Submitting Author unless you are acting as an employee on behalf of your employer or a postgraduate student of an affiliated institution which is paying any applicable article publishing charge ("APC") for Open Access articles. Where the Submitting Author wishes to make the Work available on an Open Access basis (and intends to pay the relevant APC), the terms of reuse of such Open Access shall be governed by a Creative Commons licence – details of these licences and which Creative Commons licence will apply to this Work are set out in our licence referred to above.

Other than as permitted in any relevant BMJ Author's Self Archiving Policies, I confirm this Work has not been accepted for publication elsewhere, is not being considered for publication elsewhere and does not duplicate material already published. I confirm all authors consent to publication of this Work and authorise the granting of this licence.

For peer review only

'That's how we got around it': A qualitative exploration of healthcare professionals' experiences of care provision for asylum applicants' with Limited English Proficiency in UK contingency accommodation

Corresponding author: Louise Tomkow, Humanitarian and Conflict Response Institute, Ellen Wilkinson Building, Manchester M15 6JA. louise.tomkow@manchester.ac.uk
Gabrielle Prager, Humanitarian and Conflict Response Institute, University of Manchester, Manchester, UK lgprager@doctors.org.uk
Jessica Drinkwater - Centre for Primary Care and Health Services Research, The University of Manchester, Manchester, UK Jessica.drinkwater@manchester.ac.uk
Rebecca Morris NIHR Greater Manchester Patient Safety Translational Research Centre, Centre for Primary Care and Health Services Research, Manchester Academic Health Science Centre, The University of Manchester, UK
Rebecca Farrington, Division of Medical Education, University of Manchester, Manchester, UK. GP with a Special Interest Asylum Seeker Mental Health, Greater Manchester Mental Health Foundation Trust,

For peer review only

Abstract

Objectives
The inadequate provision of language interpretation for people with Limited English Proficiency (LEP) is a determinant of poor health, yet interpreters are underused. This research explores the experiences of NHS staff providing primary care for people seeking asylum, housed in contingency accommodation during COVID-19. This group often have LEP and faces multiple additional barriers to healthcare access. Language discrimination is used as a theoretical framework. The potential utility of this concept is explored as a way of understanding and addressing inequities in care.

Design
Qualitative research using semi-structured interviews and inductive thematic analysis.

Setting
An NHS primary care service for people seeking asylum based in contingency accommodation during COVID-19 housing superdiverse residents speaking a wide spectrum of languages.

Participants
Ten staff including doctors, nurses, mental health practitioners, health care assistants and students participated in semi-structured online interviews. Some staff were redeployed to this work due to the pandemic.

Results
All interviewees described patients' LEP as significant. Inadequate provision of interpretation services impacted the staff's ability to provide care and compromised patient safety. Discrimination, such as that

based on migration status, was recognised and challenged by staff. However, inequity based on language was not articulated as discrimination. Instead, insufficient and substandard interpretation was accepted as the status quo and workarounds used, such as gesticulating or translation phone apps. The theoretical lens of language discrimination shows how this propagates existing social hierarchies and further disadvantages those with LEP.

Conclusions
This research provides empirical evidence of how the inadequate provision of interpreters forces the hand of healthcare staff to use shortcuts. Although this innovative 'tinkering' allows staff to get the job done, it risks normalising structural gaps in care provision for people with LEP. Policymakers must rethink their approach to interpretation provision which prioritises costs over quality. We assert that the concept of language discrimination is a valuable framework for clinicians to better identify and articulate unfair treatment on the grounds of LEP.

Strengths and limitations

- There is no existing research exploring UK healthcare professionals' experiences of working with asylum seekers with limited English proficiency
- We are the first to use the theoretical framework of language discrimination in a UK healthcare setting and show how it might be of value in improving care
- The qualitative data collected is in depth and rich
- The sample size is modest, due to the specialist nature of the service
- Our research design stakeholder group was made up of healthcare professionals. Future research in this area should involve patient and public contributors

For peer review only

Introduction

Good-quality communication between healthcare providers and patients is crucial for universal access to safe person-centred care.[1] In English-speaking countries worldwide, including Australia, USA and the UK, patients with Limited English Proficiency (LEP) experience barriers accessing care;[2,3] more medical errors;[4,5] lower satisfaction;[6] and misunderstanding of health information.[7] The benefits of providing high-quality interpretation include: improved clinical care;[8,9] reduced inequalities in healthcare access;[10] and cost-savings through decreased hospital readmissions and length of stay.[11,12] Despite this, interpretation services remain underused.[13] This is justified by time constraints, lack of availability of interpreters, and dissatisfaction with interpretation quality.[13] Worryingly, a study exploring doctors' reasoning suggested that a clinician-centred approach to communication might be contributory - for some doctors, communication was seen merely as a way of collecting information.[14]

Addressing population health inequalities is touted as a priority for the UK's NHS and is foregrounded in pandemic recovery policy.[15,16] NHS England outlines key principles for the provision of high-quality interpretation and translation services.[17] However, a recent UK review identified that longstanding ethnic inequalities in access, experience and healthcare outcomes are due, in part, to a lack of appropriate interpretation services.[18] Despite care providers' duty to provide interpretation services, research shows this is highly variable in practice according to service users. Family members are often used to fill the gap, with implications for patients' confidentiality [2,17,19,20]. Although we know the harmful effects of poor communication on care outcomes, there is scarce research focusing on clinicians' experiences and negotiation of language barriers.

Research context

This paper examines the experiences of NHS staff working with people with LEP in UK asylum contingency accommodation during the COVID-19 pandemic. This accommodation housed people seeking asylum from multiple countries, i.e. engaged in the lengthy legal process of applying for refugee status. A refugee is a person who 'owing to a well-founded fear of being persecuted for reasons of race, religion, nationality, membership of a particular social group, or political opinion, is outside the country of his nationality, and is unable to or, owing to such fear, is unwilling to avail himself of the protection of that country' [21]. Whilst awaiting a decision on refugee status individuals are usually housed in dispersal accommodation. Contingency accommodation - such as hotels or army barracks - is used for extra capacity. Over the last decade the UK government introduced policies purposefully making life difficult for migrants to reduce numbers. These policies and practices, often described as the "hostile environment", embed immigration controls into public services [22].

People seeking asylum face multiple obstacles accessing healthcare and frequently have diverse and additional needs to settled populations. [2] Immigration controls present in UK welfare and healthcare systems include restrictions to entitlements for free NHS care. Since 2017, certain migrants are ineligible for non-emergency care and must pay before receiving treatment. Although GP services remain free for

For peer review only - http://bmjopen.bmj.com/site/about/guidelines.xhtml

all, there is confusion amongst NHS care providers about charging and people seeking asylum often lack awareness of how to navigate the NHS.[23] Inadequate interpretation is a significant barrier to care.[24] The time-limited structure of NHS consultations can be inadequate to address complex needs.[25]

The use of contingency accommodation increased considerably in the COVID-19 pandemic [26] amplifying the difficulties of healthcare access and living conditions. Pre-pandemic healthcare access for people seeking asylum housed this way was documented as inadequate; evidence suggests this has deteriorated significantly and concerns continue to be raised regarding unsafe conditions.[27] During the pandemic, many NHS services adopted remote working, worsening existing barriers to access and communication.[28] In recognition, some primary care providers established extra services focusing on health needs in contingency accommodation.[29] This paper examines NHS staff experiences at one such service, rapidly implemented in 2020, delivering face-to-face primary care on-site for over 200 people seeking asylum. Many of the staff were redeployed as part of the COVID-19 response. Interpretation for superdiverse residents who spoke a multitude of languages was from a mix of untrained informal contacts, community volunteers, bilingual practitioners, and trained professional on-demand telephone interpreters. It was of variable quality and availability.

Research aims

This research aims to investigate the experiences of NHS staff during COVID-19, examining how staff understood, experienced and negotiated care provision for people seeking asylum with LEP. As a secondary aim, we explore the potential utility of the concept of language discrimination as a way of comprehending inequities in access to care.

Theoretical framework

We use the concept of language discrimination as a theoretical framework.[30] We understand this concept as articulated by Lippi Green: a sociological theoretical lens to observe how discrimination on the grounds of LEP perpetuates inequitable treatment and reinforces normative power hierarchies. We do so from an anti-racist position that embraces naming discrimination, then asking 'how is it operating here?' as the first steps to addressing it. [31]

Lippi-Green's work examines how discrimination on the grounds of accent or LEP benefits those who speak English as a first language over those with LEP[30]. US studies have shown how language discrimination causes individuals to feel overlooked and inferior [32] and is associated with adverse health outcomes [33]. There is little exploration of how it operates beyond the USA. In UK healthcare this scarcity of work describing language discrimination is perhaps related to opacity over the legal protection offered to those with LEP. It is not considered a 'protected characteristic' under the Equality Act 2010. However, both socially and legally, language is inextricably intertwined with nationality and race, which are protected characteristics.[33,34,35,36] As a result, NHS England's interpretation commissioning guidance acknowledges discrimination on the basis of national origin through a lack of language assistance for LEP

For peer review only

persons.[17] Consequently, we view it as an essential strategic consideration in reducing health inequalities.

We conceptualise language discrimination as both structural – created and normalised in mainstream institutions - and structuring – in that it reinforces existing hierarchies of privilege, restricting the opportunities of already-disadvantaged groups. The inadequate provision of interpretation services epitomises the operationalisation of language discrimination in healthcare and is entangled in the UK context of a hostile environment. It is important to note that adequate provision of interpreters alone would not eradicate language discrimination, as cultural humility remains important. However, for the purpose of this paper, we use adequate interpretation provision as the bare minimum, thus a proxy marker of language discrimination, using it as an empirical signal to explore the issue.

Methods

Study design

Twenty-seven healthcare professionals currently or previously working with the asylum seeker primary care service in contingency accommodation were invited via email to participate in an online interview about their experiences. In 2021, ten of these healthcare professionals participated in remote semi-structured qualitative interviews, lasting between 45 minutes and 2.5 hours. Participants included doctors, nurses, mental health practitioners, health care assistants and students, some of whom had been redeployed to primary care in the pandemic. Interviews were conducted using Zoom video conferencing software by author GP, a clinical academic with no prior relationship to the invited healthcare professionals. Interviews began with verbal consent and participants shared a brief explanation of their professional background and role. Participants were told that the research team were interested in migrant health and their experiences working at the service during COVID-19. Interviews were conversational, with open questioning using an interview guide on the practical and ethical issues of care provision in contingency accommodation. They allowed opportunities for participants to discuss issues and experiences important to them. The interview guide was developed in collaboration with health professionals experienced in migrant health work. Audio of the interviews was recorded, notes taken by the interviewer, and recordings transcribed verbatim by GP. No payment was offered for participation. Data was anonymised, with place names changed and pseudonyms to protect participants. Demographic details for individual participants are not shared due to the modest number of participants, the specialist nature of the service, and to maintain confidentiality. Two site visits were undertaken by researchers (LT and GP) to familiarise with the environment.

Data analysis

Using inductive thematic analysis, LT and GP coded data using NVivo 12 to increase robustness.[33] Dominant themes were derived from the transcripts before data was coded and extracted. First phase analysis focussed on the moral implications of care in contingency accommodation and is explored elsewhere.[37] Language and discrimination appeared as key themes and prompted additional examination. Secondary thematic analysis was therefore applied to the data using Lippi-Green's language discrimination as a theoretical framework (by GP, LT, JD, RM and RF). This occurred after data collection

For peer review only - http://bmjopen.bmj.com/site/about/guidelines.xhtml

as specific questions about language and discrimination were not in the interview topic guide. The University of Manchester Research Ethics Committee gave approval (NHS001845).

Research team and reflexivity

The research team are clinical academics with an interest in migrant health. LT (PI) and RF designed and led the research. LT developed study materials and ethics application. LT supervised GP in recruitment and interviews. All authors (LT, GP, JD, RM and RF) contributed to the analysis. Our interest in migrant health risks the imposition of our own beliefs on the research. LT and RF work in migrant health advocacy which facilitated access to the participants but could also shape both the data interpretation and the ideas in this paper. The clinical identity of the interviewer (GP) may have promoted a social desirability bias from participants. In mitigation, the interview topic guide was developed with several clinicians.

Patient and Public Involvement: None

Results

All participants stressed the importance of patients' LEP and the inadequate provision of interpretation services on care provision. Many commented on how patients were disadvantaged on the grounds of language and how this compromised patient safety. However, unlike other forms of discrimination such as racism, the disadvantage faced by people with LEP was rarely described in terms of discrimination. Instead, staff responded by improvising, using workarounds to get the job done. To illustrate, we present three emergent themes from the data: (i) recognising and resisting discrimination; (ii) the importance of interpretation provision for safe care and; (iii) improvisation around inadequate interpretation

(i) Recognising and resisting discrimination

Healthcare staff readily recognised that people seeking asylum encountered discrimination on the grounds of migration status, nationality, and race:

> *'They will face a lot of discrimination in the quality [of care] … It's been a rude awakening … there's a lot of prejudice and stereotypes of those seeking asylum.'* Participant 7

Many noticed discrimination towards their patients from healthcare staff in other settings such as pharmacy, hospital, and social care. Staff reported inappropriate questioning of patients' entitlement to NHS treatments and social care by service providers, resulting in some being denied care which they had the right to receive. Several portrayed how a dental surgery had refused to see residents from contingency accommodation and labelled this as discriminatory. One participant used these allegations of discrimination as a way of challenging dental surgery staff and advocating for all the residents:

For peer review only - http://bmjopen.bmj.com/site/about/guidelines.xhtml

> *'I said, "Can I just get this straight? So, you're telling me that one individual was abusive towards your staff, and because of that, you're going to be discriminating against 250 other individuals because you're deciding that everybody who is seeking asylum must be the same and is going to behave like this one person. Is that what you're saying?" … "No, no, no. That's not what we're saying." "Okay, good. Because that would have been discrimination, wouldn't it." These are the conversations that we have to have all of the time.'* Participant 1

This illustrates how recognising inequity as discrimination - and articulating it as such - allows individuals to challenge and resist inequitable treatment. Importantly, it demonstrates that some healthcare staff also recognise calling out discrimination as part of their role.

(ii) The importance of interpretation provision for safe care

When asked about wider experiences working in the contingency accommodation, all participants spontaneously stressed the importance of LEP and contrasted it with their previous NHS work. They acknowledged how LEP compounded the multiple complex needs of their asylum-seeking patients and the importance of language interpretation services to reduce inequity:

> *'I mean, in my view, [translation services] should be a bare minimum for any service delivering health care in the UK ... Because otherwise, you create instant inequalities. Particularly within this very, very vulnerable group of people. Why that isn't a national standard? I don't know.'* Participant 1

> *'It's quite chaotic … The difference, obviously the patient group have a lot of needs that need to be addressed and to be managed, especially the fact that English is not their first language.'* Participant 7

Participants frequently associated inadequate interpretation provision with care inequities, even highlighting safety concerns, such as COVID-19 transmission risk. This illustrates the wider public health implications of the derisory provision of information to patients with LEP:

> *'In the really early days when they were not providing information about COVID in their own languages, a lot of them didn't know what COVID was'.* Participant 10

Participant 10 draws attention to the risks posed by medication errors:

> *'The patients here didn't speak English. They can't read road signs, they don't know where the GP practice is, or the pharmacy is. And when they get to the pharmacy, they can't communicate with the lady behind the desk, so it was really difficult in that sense, and then they would get the medication and be like, "I can't read the label. How do I take this?" And then there are safety*

For peer review only - http://bmjopen.bmj.com/site/about/guidelines.xhtml

aspects to that in that they are not ingesting their medication in the right way and things like that, so that was difficult, so things that are normally easy are really, really hard.' Participant 10

This illustrates multiple structural barriers faced by patients with LEP trying to navigate health systems, access care and self-manage. It demonstrates the need for improvements in interpretation services beyond medical consultations, including translation of signage, pamphlets and labels. LEP instigates barriers to accessing safe care and achieving good health not encountered by English speakers, showing how language discrimination propagates existing social hierarchies. Although participants recognised structured and structuring aspects of inadequate interpretation provision, they did not articulate it as discriminatory.

(iii) Improvisation around inadequate interpretation

All participants reported facing challenges accessing adequate interpretation services. Many described the resulting suboptimal care. As part of the pandemic response, volunteers from local refugee charities initially provided face-to-face interpretation at the contingency accommodation for medical consultations. This in-person service was described by Participant 2 as '*so useful … made life so much easier … made such a difference'*. However, this was impossible beyond the first few months as their own voluntary organisations reopened. The clinical team were left using telephone interpretation services, universally reported as problematic and often labelled a *'waste'* of time:

> *'The waits for [the telephone interpretation service] were quite extensive. 30 minutes, 45 minutes, which had a huge impact on clinical time. And you would often get through and then not be able to get through to the patient. We were wasting a huge number of clinical hours at that point in just trying to get hold of patients and speak to them.'* Participant 1

The poor mobile phone signal in the accommodation caused frequent and lengthy interruptions. When staff were able to connect, the quality of interpretation was variable and often substandard:

> *'Someone would talk at length sometimes and the interpreter would summarise it in a few sentences, so I don't know if I was actually getting the full picture … sometimes because [patients] had partial English they would sometimes say 'I'm not saying that'.'* Participant 9

Inadequate interpretation services in secondary care impacted the workload of the primary care participants. Staff in an already-pressured environment took time to fill the gap left by inadequate language provision:

> *'The doctor sends the referrals, and then the patient gets a letter, but they can't read the letter in English. So, a lot of my job was reading the letter to an interpreter on the phone and explaining it to the patient … Or telephone appointments without times so we can't organise a translator'* Participant 2

For peer review only - http://bmjopen.bmj.com/site/about/guidelines.xhtml

Doubts about the quality of interpretation and an imperative to use time efficiently resulted in some participants avoiding telephone interpretation. Instead, they improvised with non-verbal cues, gesticulations and mobile phone apps. This approach is evocative of 'tinkering', described by Mol in 2008 [38], as a way of adjusting towards situationally-determined improved outcomes:

> *'I think I was very much like, I don't want to use this unless I really have to … I tried to communicate like, with facial expressions and hand gestures'* Participant 5

> *'we just didn't have the time [to use telephone interpretation] … we started to use the phone app … I'm sure that it affected care.'* Participant 2

Recognising the importance of good communication for safe care, participants were driven to search for workarounds in the face of limited time, resources and poor-quality interpretation services. Many 'tinkered' to moderate and simplify communication:

> *'it's about how to simplify the language and talk more slowly and talk in shorter sentences because I was going through a translator'* Participant 9

> *'being resourceful, i.e. drawing pictures on paper, using body language or hand gestures as signals and things like that. It is not always easy because of COVID, and you are wearing a face mask … sometimes I use Google Translate or 'Siri' will speak.' Participant 10*

Although some framed this innovation in a positive light, others suggested how this might compromise care:

> *'[some people] they would translate for their friends, which again is an issue with confidentiality and safety at times. I'm not sure if I would want to disclose some things to my friends. That was a last resort … I guess things could have been missed if people didn't want to disclose some information, but that's how we got around it'* Participant 2

Again, the insufficient provision of interpretation was not articulated as discrimination.

For peer review only - http://bmjopen.bmj.com/site/about/guidelines.xhtml

Discussion

This qualitative analysis illustrates how staff understood the disadvantage experienced by those with LEP differently from that based on migration status or ethnicity. When discrimination based on migration status was identified, some staff saw calling it out as part of their role and successfully facilitated change. In contrast, the impact of inadequate interpretation on providing safe and trauma-informed care was not identified as discriminatory, despite all of those interviewed recognising the risks. Consequently, it was not challenged in the same way. Instead, when faced with a lack of available face-to-face interpreters and poor-quality difficult-to-access telephone interpretation, staff 'tinkered', using workarounds such as translation apps, gesticulating or drawings.

The concept of 'tinkering' has been used to explore care practices in various resource-poor contexts.[38,39]. Recently, ethnographic work has demonstrated the resourcefulness of healthcare staff and the necessity of creative innovations in low-income settings: "'[i]f the system doesn't work, you have to make it work for you, for the sake of the patient'".[40] However, tinkering has limits - Reider describes how it can lead to physician disenfranchisement and departure.[41] By framing these practices through the lens of language discrimination, we add to this critical perspective on the practice of tinkering. When staff feel workarounds are their only option, it seems that 'tinkering' can become usual practice, even in resource-rich countries where healthcare faces ideologically driven cuts. Inequalities are thus reproduced, normalised, and embedded within organisational structures and the ability to change discriminatory systems is diminished.

US-based research links the concept of language discrimination and patients' poor experiences of healthcare access.[42,43] However, we appear to be the first to apply the sociological concept of language discrimination to the UK healthcare setting, use it to critically analyse healthcare providers' narratives of care provision, and argue for its more widespread application as a means to address inequalities. In doing so, we make an important theoretical contribution. Empirically, language discrimination is evident throughout this data: differential treatment on the grounds of LEP perpetuates inequalities in care and maintains the normative hierarchies of power and privilege, where people who speak English receive better care. We caution against the assumption that language is a modifiable characteristic and therefore not challenged as readily as other forms of discrimination, such as racism. This speculation overlooks the well-described structural, psychological, social and educational barriers to learning new languages. [44]

Anti-racist scholars assert that naming discrimination and examining how it operates are the first steps in confronting inequitable health systems.[31] We uphold that promoting the application of the concept of language discrimination is the first step in addressing inequitable care for those with LEP. Naming inadequate interpretation provision in healthcare as a form of discrimination works to overcome institutional and social inertia, empowering staff and giving them vocabulary to challenge health inequalities experienced by people with LEP. In the contemporary UK context, the gap in interpretation provision can be understood as part of a broader discriminatory approach to migrants. Technical solutions to improve care are needed, with provision of culturally-sensitive interpreters as the bare minimum.

For peer review only - http://bmjopen.bmj.com/site/about/guidelines.xhtml

Moreover, language discrimination is only one facet of the hostile environment facing migrants in the UK (22). Since undertaking this research, living conditions for people seeking asylum have been further eroded, with plans for offshore processing and accommodation on sea vessels.

Illuminating the issues from a practitioner perspective, this paper contributes to the literature on the underuse of interpreters in healthcare.[14] We emphasise the chasm between academic knowledge, assertions from policymakers, and the reality of conditions facing practitioners. A wealth of literature documents the harms of inadequate interpretation alongside healthcare policy proclaiming that those with LEP should not experience worse care.[16,17] Yet when healthcare staff face high clinical workloads without access to user-friendly high-quality interpretation, using workarounds to get the job done becomes usual practice and inequity becomes institutionalised. It is important to note that in countries where payment is structured differently the extra time required for interpretation may also impact clinician income.

Our empirical findings illuminate why health outcomes are pitiful when provision of interpretation services is inadequate and shed light on the variable uptake of healthcare services across diverse communities. They should interest practitioners and researchers concerned with structural causes of health inequalities. Our analysis highlights that some social groups are underserved rather than 'difficult to reach'. Of relevance for policymakers is the lack of transparency around commissioning for interpretation provision in England and the marked variability in expenditure for comparable demographics.[45,46] A radical overhaul of interpretation provision is crucial to address ethnic inequalities in health.

This paper focuses on language discrimination, a previously under-explored yet important component of ethnic health inequalities. The qualitative data is rich and extensive; however, the study does have limitations. The number of participants is modest due to the specialist nature of the service. There may be social desirability bias of participants' self-representation to the interviewer, who was a clinician and researcher. These biases could arguably make the findings more salient. The staff at this specialist service often went above and beyond usual care and it is likely that the normalisation and institutionalisation identified here would be readily replicated elsewhere by less committed individuals. Research exploring how these findings translate to other settings would be valuable.

Conclusion

This research provides empirical evidence of how the inadequate provision of interpreters, shown here in the context of the pandemic, appears to force the hand of healthcare staff to use shortcuts. This risks compromising the safe care of people with LEP. Both structural and operational changes are needed to improve healthcare provision for migrants. Inadequate interpretation provision should be understood as part of a complex system of discrimination facing people seeking asylum which requires a radical overhaul. More specifically, policymakers must rethink their approach to interpretation provision

For peer review only - http://bmjopen.bmj.com/site/about/guidelines.xhtml

including training of practitioners to work with interpreters, integrating systems to reduce factors that prevent their use, and prioritising quality over cost to ensure safety.

Endorsement of the concept of language discrimination in healthcare would provide a useful framework for clinicians to better identify and articulate unfair treatment on the grounds of LEP. Borrowing from anti-racist scholarship, we argue that this is the first step in shifting the culture around the ubiquitous but hidden acceptance of a poor standard of care for LEP patients. Staff were tenacious and challenged practices where more overt examples of discrimination were experienced and could be named. Future research should examine healthcare workers' perception of the concept of language discrimination and the utility and limitations of the concept in addressing health inequalities.

For peer review only

References

1. Ratna H. The importance of effective communication in healthcare practice. Harvard Public Health Review. 2019 Oct 1;23:1-6.
2. Kang C, Tomkow L, Farrington R. Access to primary health care for asylum seekers and refugees: a qualitative study of service user experiences in the UK. British Journal of General Practice. 2019 Aug 1;69(685):e537-45.
3. White J, Plompen T, Osadnik C, Tao L, Micallef E, Haines T. The experience of interpreter access and language discordant clinical encounters in Australian health care: a mixed methods exploration. International journal for equity in health. 2018 Dec;17(1):1-0.
4. Divi C, Koss RG, Schmaltz SP, Loeb JM. Language proficiency and adverse events in US hospitals: a pilot study. International journal for quality in health care. 2007 Apr 1;19(2):60-7.
5. Flores G, Laws MB, Mayo SJ, Zuckerman B, Abreu M, Medina L, Hardt EJ. Errors in medical interpretation and their potential clinical consequences in pediatric encounters. Pediatrics. 2003 Jan;111(1):6-14.
6. Baker DW, Hayes R, Fortier JP. Interpreter use and satisfaction with interpersonal aspects of care for Spanish-speaking patients. Medical care. 1998 Oct 1:1461-70.
7. Wilson E, Chen AH, Grumbach K, Wang F, Fernandez A. Effects of limited English proficiency and physician language on health care comprehension. Journal of general internal medicine. 2005 Sep;20(9):800-6.
8. Karliner LS, Jacobs EA, Chen AH, Mutha S. Do professional interpreters improve clinical care for patients with limited English proficiency? A systematic review of the literature. Health services research. 2007 Apr;42(2):727-54.
9. Lee JS, Pérez-Stable EJ, Gregorich SE, Crawford MH, Green A, Livaudais-Toman J, Karliner LS. Increased access to professional interpreters in the hospital improves informed consent for patients with limited English proficiency. Journal of general internal medicine. 2017 Aug;32(8):863-70.
10. Jacobs EA, Lauderdale DS, Meltzer D, Shorey JM, Levinson W, Thisted RA. Impact of interpreter services on delivery of health care to limited-English-proficient patients. Journal of general internal medicine. 2001 Jul;16(7):468-74.

11. Karliner LS, Pérez-Stable EJ, Gregorich SE. Convenient access to professional interpreters in the hospital decreases readmission rates and estimated hospital expenditures for patients with limited English proficiency. Medical care. 2017 Mar;55(3):199.
12. John-Baptiste A, Naglie G, Tomlinson G, Alibhai SM, Etchells E, Cheung A, Kapral M, Gold WL, Abrams H, Bacchus M, Krahn M. The effect of English language proficiency on length of stay and in-hospital mortality. Journal of general internal medicine. 2004 Mar;19(3):221-8.
13. Kale E, Syed HR. Language barriers and the use of interpreters in the public health services. A questionnaire-based survey. Patient education and counseling. 2010 Nov 1;81(2):187-91.
14. Diamond LC, Schenker Y, Curry L, Bradley EH, Fernandez A. Getting by: underuse of interpreters by resident physicians. Journal of general internal medicine. 2009 Feb;24(2):256-62.
15. NHS Long term plan, NHE England. Available at: https://www.england.nhs.uk/long-term-plan/ Accessed 13.10.22
16. Ethnic Inequalities in Healthcare: A Rapid Evidence Review. NHS race and health observatory. Available at: https://www.nhsrho.org/wp-content/uploads/2022/02/RHO-Rapid-Review-Final-Report_Summary_v.4.pdf Accessed 13.10.22
17. Guidance for commissioners interpreting and translation services in primary care, NHS England. Available at: https://www.england.nhs.uk/wp-content/uploads/2018/09/guidance-for-commissioners-interpreting-and-translation-services-in-primary-care.pdf Accessed 13.10.22
18. Patel KC, Hanif W. Ethnic health inequalities in the NHS. BMJ. 2022 Mar 9;376.
19. Barron, D.S., Holterman, C., Shipster, P., Batson, S. and Alam, M., 2010. Seen but not heard–ethnic minorities' views of primary health care interpreting provision: a focus group study. *Primary Health Care Research & Development*, *11*(2), pp.132-141.
20. Gill, P.S., Beavan, J., Calvert, M. and Freemantle, N., 2011. The unmet need for interpreting provision in UK primary care. *PLoS one*, *6*(6), p.e20837.
21. https://www.unhcr.org/uk/asylum-uk
22. Griffiths, M. and Yeo, C., 2021. The UK's hostile environment: Deputising immigration control. *Critical Social Policy*, *41*(4), pp.521-544.
23. Tomkow LJ, Kang CP, Farrington RL, Wiggans RE, Wilson RJ, Pushkar P, Tickell-Painter MC, Lee AR, Whitehouse ER, Mahmood NG, Lawton KM. Healthcare access for asylum seekers and refugees in England: a mixed methods study exploring service users' and health care professionals' awareness. European Journal of Public Health. 2020 Jun 1;30(3):527-32.
24. Patel P, Bernays S, Dolan H, Muscat DM, Trevena L. Communication experiences in primary healthcare with refugees and asylum seekers: a literature review and narrative synthesis. International journal of environmental research and public health. 2021 Feb;18(4):1469.
25. Arnold FW. How can we help asylum seekers get better healthcare?. bmj. 2022 Feb 7;376.
26. The Home Office. The use of temporary hotels to house asylum seekers during Covid-19. August 2020. Available at: https://homeofficemedia.blog.gov.uk/2020/08/08/the-use-of-temporary-hotels-to-house-asylum-seekers-during-covid-19/ Accessed August 2022
27. Jones L, Phillimore J, Fu L, Hourani J, Lessard-Phillips L, Tatem B. Doctors of the World Report. *They just left me Asylum seekers, health, and access to healthcare in initial and contingency accommodation. 2022.* Report. Available at: https://www.doctorsoftheworld.org.uk/wp-

For peer review only - http://bmjopen.bmj.com/site/about/guidelines.xhtml

content/uploads/2022/04/DOTW-Access-to-healthcare-in-initial-and-contingency-accommodation-report-April-2022.pdf. Accessed March 2023
28. Written evidence submitted by Doctors of the World (DOTW) (COR0203) [Internet]. [cited 2022 Mar 28]. Available from: https://bjgp.org/content/69/685/e537#ref-6
29. Farrant O, Eisen S, Van Tulleken C, Ward A, Longley N. Why asylum seekers deserve better healthcare, and how we can give it to them. bmj. 2022 Jan 6;376.
30. Lippi-Green R. English with an accent: Language, ideology, and discrimination in the United States. Routledge; 2012 Mar 15.
31. Jones CP. Confronting institutionalized racism. Phylon (1960-). 2002 Jan 1:7-22.
32. Lee JJ, Rice C. Welcome to America? International student perceptions of discrimination. Higher education. 2007 Mar;53(3):381-409.
33. Yoo HC, Gee GC, Takeuchi D. Discrimination and health among Asian American immigrants: Disentangling racial from language discrimination. Social science & medicine. 2009 Feb 1;68(4):726-32.
34. Language requirements can be a legal minefield. Employment Law. Available at:
35. https://www.peoplemanagement.co.uk/article/1745542/language-requirements-legal-minefield Accessed 13.10.22
36. Core20PLUS5 – An approach to reducing health inequalities. Available at: https://www.england.nhs.uk/about/equality/equality-hub/national-healthcare-inequalities-improvement-programme/core20plus5/#:~:text=Core20PLUS5%20is%20a%20national%20NHS,clinical%20areas%20requiring%20accelerated%20improvement Accessed 9.11.22
37. Tomkow, L., Prager, G., Worthing, K. and Farrington, R., 2023. In critique of moral resilience: UK healthcare professionals' experiences working with asylum applicants housed in contingency accommodation during the COVID-19 pandemic. *Journal of Medical Ethics*.
38. Mol, A., 2008. *The logic of care: Health and the problem of patient choice*. Routledge.
39. Trundle, C., 2020. Tinkering care, state responsibility, and abandonment: nuclear test veterans and the mismatched temporalities of justice in claims for health care. *Anthropology and Humanism*, *45*(2), pp.202-211.
40. Vernooij, E., Koker, F. and Street, A., 2022. Responsibility, repair and care in Sierra Leone's health system. *Social Science & Medicine*, *300*, p.114260.
41. Rieder, S., 2017. Tinkering toward departure: The limits of improvisation in rural Ethiopian biomedical practices. *Social Science & Medicine*, *179*, pp.1-8.
42. Weng, S.S. and Wolfe III, W.T., 2016. Asian American health inequities: An exploration of cultural and language incongruity and discrimination in accessing and utilizing the healthcare system. *International Public Health Journal*, *8*(2).
43. Halim, M.L., Moy, K.H. and Yoshikawa, H., 2017. Perceived ethnic and language-based discrimination and Latina immigrant women's health. *Journal of Health Psychology*, *22*(1), pp.68-78.
44. Thomas DR. A general inductive approach for analyzing qualitative evaluation data. American journal of evaluation. 2006 Jun;27(2):237-46.

For peer review only - http://bmjopen.bmj.com/site/about/guidelines.xhtml

45. Morrice L, Tip LK, Collyer M, Brown R. 'You can't have a good integration when you don't have a good communication': English-language learning among resettled refugees in England. Journal of Refugee Studies. 2021 Jun 26;34(1):681-99.
46. Lehane D, Oliver P, Mitchell C, Burton C. Interpreter costs across clinical commissioning groups in England 2017–2018: a cross sectional survey using 'freedom of information'requests. British Journal of General Practice. 2020 Jun 1;70(suppl 1).

COMPETING INTERESTS

None

FUNDING

No specific funding to report for this work. GP is an Academic Clinical Fellow and LT and JD are Clinical Lecturers funded by Health Education England (HEE) and NIHR. RM's input was supported by the National Institute for health and care Research (NIHR) Greater Manchester Patient Safety Translational Researcg Centre (award number PSTRC-2016-003). The views expressed in this publication are those of the author(s) and not necessarily those of the NIHR, NHS or the UK Department of Health and Social Care.

For peer review only

For peer review only - http://bmjopen.bmj.com/site/about/guidelines.xhtml

Standards for Reporting Qualitative Research (SRQR)*

http://www.equator-network.org/reporting-guidelines/srqr/

Title and abstract

	Page/line no(s).
**Title** - Concise description of the nature and topic of the study Identifying the study as qualitative or indicating the approach (e.g., ethnography, grounded theory) or data collection methods (e.g., interview, focus group) is recommended	Page 1 Line 1
**Abstract** - Summary of key elements of the study using the abstract format of the intended publication; typically includes background, purpose, methods, results, and conclusions	Page 1 lines 3 onwards

Introduction

**Problem formulation** - Description and significance of the problem/phenomenon studied; review of relevant theory and empirical work; problem statement	Pages 3-5
**Purpose or research question** - Purpose of the study and specific objectives or questions	Pages 3-5

Methods

**Qualitative approach and research paradigm** - Qualitative approach (e.g., ethnography, grounded theory, case study, phenomenology, narrative research) and guiding theory if appropriate; identifying the research paradigm (e.g., postpositivist, constructivist/ interpretivist) is also recommended; rationale**	Pages 5-6
**Researcher characteristics and reflexivity** - Researchers' characteristics that may influence the research, including personal attributes, qualifications/experience, relationship with participants, assumptions, and/or presuppositions; potential or actual interaction between researchers' characteristics and the research questions, approach, methods, results, and/or transferability	Page 6
**Context** - Setting/site and salient contextual factors; rationale**	Pages 3, 5-6
**Sampling strategy** - How and why research participants, documents, or events were selected; criteria for deciding when no further sampling was necessary (e.g., sampling saturation); rationale**	Page 5
**Ethical issues pertaining to human subjects** - Documentation of approval by an appropriate ethics review board and participant consent, or explanation for lack thereof; other confidentiality and data security issues	Page 6
**Data collection methods** - Types of data collected; details of data collection procedures including (as appropriate) start and stop dates of data collection and analysis, iterative process, triangulation of sources/methods, and modification of procedures in response to evolving study findings; rationale**	Pages 5-6

For peer review only - http://bmjopen.bmj.com/site/about/guidelines.xhtml

For peer review only

**Data collection instruments and technologies** - Description of instruments (e.g., interview guides, questionnaires) and devices (e.g., audio recorders) used for data collection; if/how the instrument(s) changed over the course of the study	Pages 5-6
**Units of study** - Number and relevant characteristics of participants, documents, or events included in the study; level of participation (could be reported in results)	Pages 5-6
**Data processing** - Methods for processing data prior to and during analysis, including transcription, data entry, data management and security, verification of data integrity, data coding, and anonymization/de-identification of excerpts	Pages 5-6
**Data analysis** - Process by which inferences, themes, etc., were identified and developed, including the researchers involved in data analysis; usually references a specific paradigm or approach; rationale**	Pages 5-6
**Techniques to enhance trustworthiness** - Techniques to enhance trustworthiness and credibility of data analysis (e.g., member checking, audit trail, triangulation); rationale**	Pages 5-6

Results/findings

**Synthesis and interpretation** - Main findings (e.g., interpretations, inferences, and themes); might include development of a theory or model, or integration with prior research or theory	Pages 6-11
**Links to empirical data** - Evidence (e.g., quotes, field notes, text excerpts, photographs) to substantiate analytic findings	Pages 6-11

Discussion

**Integration with prior work, implications, transferability, and contribution(s) to the field -** Short summary of main findings; explanation of how findings and conclusions connect to, support, elaborate on, or challenge conclusions of earlier scholarship; discussion of scope of application/generalizability; identification of unique contribution(s) to scholarship in a discipline or field	Pages 12-14
**Limitations** - Trustworthiness and limitations of findings	Page 13

Other

**Conflicts of interest** - Potential sources of influence or perceived influence on study conduct and conclusions; how these were managed	Page 17
**Funding** - Sources of funding and other support; role of funders in data collection, interpretation, and reporting	Page 17

*The authors created the SRQR by searching the literature to identify guidelines, reporting standards, and critical appraisal criteria for qualitative research; reviewing the reference lists of retrieved sources; and contacting experts to gain feedback. The SRQR aims to improve the transparency of all aspects of qualitative research by providing clear standards for reporting qualitative research.

For peer review only

**The rationale should briefly discuss the justification for choosing that theory, approach, method, or technique rather than other options available, the assumptions and limitations implicit in those choices, and how those choices influence study conclusions and transferability. As appropriate, the rationale for several items might be discussed together.

Reference:

O'Brien BC, Harris IB, Beckman TJ, Reed DA, Cook DA. **Standards for reporting qualitative research: a synthesis of recommendations.** *Academic Medicine*, Vol. 89, No. 9 / Sept 2014 DOI: 10.1097/ACM.0000000000000388

For peer review only

For peer review only - http://bmjopen.bmj.com/site/about/guidelines.xhtml

BMJ Open

‘That’s how we got around it’: A qualitative exploration of healthcare professionals’ experiences of care provision for asylum applicants’ with Limited English Proficiency in UK contingency accommodation

Journal:	*BMJ Open*
Manuscript ID	bmjopen-2023-074824.R2
Article Type:	Original research
Date Submitted by the Author:	06-Oct-2023
Complete List of Authors:	Tomkow, Louise; The University of Manchester,<br>Prager, Gabrielle; Johns Hopkins University<br>Drinkwater, Jessica; The University of Manchester, Centre for Primary Care and Health Services Research<br>Morris, Rebecca L.; The University of Manchester, Centre for Primary Care<br>Farrington, Rebecca; University of Manchester Faculty of Biology, Medicine and Health, Division of Medical Education
**Primary Subject Heading**:	Communication
Secondary Subject Heading:	General practice / Family practice
Keywords:	Health Services Accessibility, Primary Health Care, Health Equity, Quality in health care < HEALTH SERVICES ADMINISTRATION & MANAGEMENT, Organisation of health services < HEALTH SERVICES ADMINISTRATION & MANAGEMENT

SCHOLARONE™
Manuscripts

I, the Submitting Author has the right to grant and does grant on behalf of all authors of the Work (as defined in the below author licence), an exclusive licence and/or a non-exclusive licence for contributions from authors who are: i) UK Crown employees; ii) where BMJ has agreed a CC-BY licence shall apply, and/or iii) in accordance with the terms applicable for US Federal Government officers or employees acting as part of their official duties; on a worldwide, perpetual, irrevocable, royalty-free basis to BMJ Publishing Group Ltd ("BMJ") its licensees and where the relevant Journal is co-owned by BMJ to the co-owners of the Journal, to publish the Work in this journal and any other BMJ products and to exploit all rights, as set out in our licence.

The Submitting Author accepts and understands that any supply made under these terms is made by BMJ to the Submitting Author unless you are acting as an employee on behalf of your employer or a postgraduate student of an affiliated institution which is paying any applicable article publishing charge ("APC") for Open Access articles. Where the Submitting Author wishes to make the Work available on an Open Access basis (and intends to pay the relevant APC), the terms of reuse of such Open Access shall be governed by a Creative Commons licence – details of these licences and which Creative Commons licence will apply to this Work are set out in our licence referred to above.

Other than as permitted in any relevant BMJ Author's Self Archiving Policies, I confirm this Work has not been accepted for publication elsewhere, is not being considered for publication elsewhere and does not duplicate material already published. I confirm all authors consent to publication of this Work and authorise the granting of this licence.

For peer review only

'That's how we got around it': A qualitative exploration of healthcare professionals' experiences of care provision for asylum applicants' with Limited English Proficiency in UK contingency accommodation

Corresponding author: Louise Tomkow, Humanitarian and Conflict Response Institute, Ellen Wilkinson Building, Manchester M15 6JA. louise.tomkow@manchester.ac.uk
Gabrielle Prager, Humanitarian and Conflict Response Institute, University of Manchester, Manchester, UK lgprager@doctors.org.uk
Jessica Drinkwater - Centre for Primary Care and Health Services Research, The University of Manchester, Manchester, UK Jessica.drinkwater@manchester.ac.uk
Rebecca Morris NIHR Greater Manchester Patient Safety Translational Research Centre, Centre for Primary Care and Health Services Research, Manchester Academic Health Science Centre, The University of Manchester, UK
Rebecca Farrington, Division of Medical Education, University of Manchester, Manchester, UK. GP with a Special Interest Asylum Seeker Mental Health, Greater Manchester Mental Health Foundation Trust,

For peer review only

Abstract

Objectives

The inadequate provision of language interpretation for people with Limited English Proficiency (LEP) is a determinant of poor health, yet interpreters are underused. This research explores the experiences of NHS staff providing primary care for people seeking asylum, housed in contingency accommodation during COVID-19. This group often have LEP and faces multiple additional barriers to healthcare access. Language discrimination is used as a theoretical framework. The potential utility of this concept is explored as a way of understanding and addressing inequities in care.

Design

Qualitative research using semi-structured interviews and inductive thematic analysis.

Setting

An NHS primary care service for people seeking asylum based in contingency accommodation during COVID-19 housing superdiverse residents speaking a wide spectrum of languages.

Participants

Ten staff including doctors, nurses, mental health practitioners, health care assistants and students participated in semi-structured online interviews. Some staff were redeployed to this work due to the pandemic.

Results

All interviewees described patients' LEP as significant. Inadequate provision of interpretation services impacted the staff's ability to provide care and compromised patient safety. Discrimination, such as that

For peer review only - http://bmjopen.bmj.com/site/about/guidelines.xhtml

based on migration status, was recognised and challenged by staff. However, inequity based on language was not articulated as discrimination. Instead, insufficient and substandard interpretation was accepted as the status quo and workarounds used, such as gesticulating or translation phone apps. The theoretical lens of language discrimination shows how this propagates existing social hierarchies and further disadvantages those with LEP.

Conclusions
This research provides empirical evidence of how the inadequate provision of interpreters forces the hand of healthcare staff to use shortcuts. Although this innovative 'tinkering' allows staff to get the job done, it risks normalising structural gaps in care provision for people with LEP. Policymakers must rethink their approach to interpretation provision which prioritises costs over quality. We assert that the concept of language discrimination is a valuable framework for clinicians to better identify and articulate unfair treatment on the grounds of LEP.

Strengths and limitations

- There is no existing research exploring UK healthcare professionals' experiences of working with asylum seekers with limited English proficiency
- We are the first to use the theoretical framework of language discrimination in a UK healthcare setting and show how it might be of value in improving care
- The qualitative data collected is in depth and rich
- The sample size is modest, due to the specialist nature of the service
- Our research design stakeholder group was made up of healthcare professionals. Future research in this area should involve patient and public contributors

For peer review only

Introduction

Good-quality communication between healthcare providers and patients is crucial for universal access to safe person-centred care.[1] In English-speaking countries worldwide, including Australia, USA and the UK, patients with Limited English Proficiency (LEP) experience barriers accessing care;[2,3] more medical errors;[4,5] lower satisfaction;[6] and misunderstanding of health information.[7] The benefits of providing high-quality interpretation include: improved clinical care;[8,9] reduced inequalities in healthcare access;[10] and cost-savings through decreased hospital readmissions and length of stay.[11,12] Despite this, interpretation services remain underused.[13] This is justified by time constraints, lack of availability of interpreters, and dissatisfaction with interpretation quality.[13] Worryingly, a study exploring doctors' reasoning suggested that a clinician-centred approach to communication might be contributory - for some doctors, communication was seen merely as a way of collecting information.[14]

Addressing population health inequalities is touted as a priority for the UK's NHS and is foregrounded in pandemic recovery policy.[15,16] NHS England outlines key principles for the provision of high-quality interpretation and translation services.[17] However, a recent UK review identified that longstanding ethnic inequalities in access, experience and healthcare outcomes are due, in part, to a lack of appropriate interpretation services.[18] Despite care providers' duty to provide interpretation services, research shows this is highly variable in practice according to service users. Family members are often used to fill the gap, with implications for patients' confidentiality [2,17,19,20]. Although we know the harmful effects of poor communication on care outcomes, there is scarce research focusing on clinicians' experiences and negotiation of language barriers.

Research context

This paper examines the experiences of NHS staff working with people with LEP in UK asylum contingency accommodation during the COVID-19 pandemic. This accommodation housed people seeking asylum from multiple countries, i.e. engaged in the lengthy legal process of applying for refugee status. A refugee is a person who 'owing to a well-founded fear of being persecuted for reasons of race, religion, nationality, membership of a particular social group, or political opinion, is outside the country of his nationality, and is unable to or, owing to such fear, is unwilling to avail himself of the protection of that country' [21]. Whilst awaiting a decision on refugee status individuals are usually housed in dispersal accommodation. Contingency accommodation - such as hotels or army barracks - is used for extra capacity. Over the last decade the UK government introduced policies purposefully making life difficult for migrants to reduce numbers. These policies and practices, often described as the "hostile environment", embed immigration controls into public services [22].

People seeking asylum face multiple obstacles accessing healthcare and frequently have diverse and additional needs to settled populations. [2] Immigration controls present in UK welfare and healthcare systems include restrictions to entitlements for free NHS care. Since 2017, certain migrants are ineligible for non-emergency care and must pay before receiving treatment. Although GP services remain free for

For peer review only - http://bmjopen.bmj.com/site/about/guidelines.xhtml

all, there is confusion amongst NHS care providers about charging and people seeking asylum often lack awareness of how to navigate the NHS.[23] Inadequate interpretation is a significant barrier to care.[24] The time-limited structure of NHS consultations can be inadequate to address complex needs.[25]

The use of contingency accommodation increased considerably in the COVID-19 pandemic [26] amplifying the difficulties of healthcare access and living conditions. Pre-pandemic healthcare access for people seeking asylum housed this way was documented as inadequate; evidence suggests this has deteriorated significantly and concerns continue to be raised regarding unsafe conditions.[27] During the pandemic, many NHS services adopted remote working, worsening existing barriers to access and communication.[28] In recognition, some primary care providers established extra services focusing on health needs in contingency accommodation.[29] This paper examines NHS staff experiences at one such service, rapidly implemented in 2020, delivering face-to-face primary care on-site for over 200 people seeking asylum. Many of the staff were redeployed as part of the COVID-19 response. Interpretation for superdiverse residents who spoke a multitude of languages was from a mix of untrained informal contacts, community volunteers, bilingual practitioners, and trained professional on-demand telephone interpreters. It was of variable quality and availability.

Research aims

This research aims to investigate the experiences of NHS staff during COVID-19, examining how staff understood, experienced and negotiated care provision for people seeking asylum with LEP. As a secondary aim, we explore the potential utility of the concept of language discrimination as a way of comprehending inequities in access to care.

Theoretical framework

We use the concept of language discrimination as a theoretical framework.[30] We understand this concept as articulated by Lippi Green: a sociological theoretical lens to observe how discrimination on the grounds of LEP perpetuates inequitable treatment and reinforces normative power hierarchies. We do so from an anti-racist position that embraces naming discrimination, then asking 'how is it operating here?' as the first steps to addressing it. [31]

Lippi-Green's work examines how discrimination on the grounds of accent or LEP benefits those who speak English as a first language over those with LEP[30]. US studies have shown how language discrimination causes individuals to feel overlooked and inferior [32] and is associated with adverse health outcomes [33]. There is little exploration of how it operates beyond the USA. In UK healthcare this scarcity of work describing language discrimination is perhaps related to opacity over the legal protection offered to those with LEP. It is not considered a 'protected characteristic' under the Equality Act 2010. However, both socially and legally, language is inextricably intertwined with nationality and race, which are protected characteristics.[33,34,35,36] As a result, NHS England's interpretation commissioning guidance acknowledges discrimination on the basis of national origin through a lack of language assistance for LEP

For peer review only

persons.[17] Consequently, we view it as an essential strategic consideration in reducing health inequalities.

We conceptualise language discrimination as both structural – created and normalised in mainstream institutions - and structuring – in that it reinforces existing hierarchies of privilege, restricting the opportunities of already-disadvantaged groups. The inadequate provision of interpretation services epitomises the operationalisation of language discrimination in healthcare and is entangled in the UK context of a hostile environment. It is important to note that adequate provision of interpreters alone would not eradicate language discrimination, as cultural humility remains important. However, for the purpose of this paper, we use adequate interpretation provision as the bare minimum, thus a proxy marker of language discrimination, using it as an empirical signal to explore the issue.

Methods

Study design

Twenty-seven healthcare professionals currently or previously working with the asylum seeker primary care service in contingency accommodation were invited via email to participate in an online interview about their experiences. In 2021, ten of these healthcare professionals participated in remote semi-structured qualitative interviews, lasting between 45 minutes and 2.5 hours. Participants included doctors, nurses, mental health practitioners, health care assistants and students, some of whom had been redeployed to primary care in the pandemic. Interviews were conducted using Zoom video conferencing software by author GP, a clinical academic with no prior relationship to the invited healthcare professionals. Interviews began with verbal consent and participants shared a brief explanation of their professional background and role. Participants were told that the research team were interested in migrant health and their experiences working at the service during COVID-19. Interviews were conversational, with open questioning using an interview guide on the practical and ethical issues of care provision in contingency accommodation. They allowed opportunities for participants to discuss issues and experiences important to them. The interview guide was developed in collaboration with health professionals experienced in migrant health work. Audio of the interviews was recorded, notes taken by the interviewer, and recordings transcribed verbatim by GP. No payment was offered for participation. Data was anonymised, with place names changed and pseudonyms to protect participants. Demographic details for individual participants are not shared due to the modest number of participants, the specialist nature of the service, and to maintain confidentiality. Two site visits were undertaken by researchers (LT and GP) to familiarise with the environment.

Data analysis

Using inductive thematic analysis, LT and GP coded data using NVivo 12 to increase robustness.[33] Dominant themes were derived from the transcripts before data was coded and extracted. First phase analysis focussed on the moral implications of care in contingency accommodation and is explored elsewhere.[37] Language and discrimination appeared as key themes and prompted additional examination. Secondary thematic analysis was therefore applied to the data using Lippi-Green's language discrimination as a theoretical framework (by GP, LT, JD, RM and RF). This occurred after data collection

For peer review only - http://bmjopen.bmj.com/site/about/guidelines.xhtml

as specific questions about language and discrimination were not in the interview topic guide. The University of Manchester Research Ethics Committee gave approval (NHS001845).

Research team and reflexivity

The research team are clinical academics with an interest in migrant health. LT (PI) and RF designed and led the research. LT developed study materials and ethics application. LT supervised GP in recruitment and interviews. All authors (LT, GP, JD, RM and RF) contributed to the analysis. Our interest in migrant health risks the imposition of our own beliefs on the research. LT and RF work in migrant health advocacy which facilitated access to the participants but could also shape both the data interpretation and the ideas in this paper. The clinical identity of the interviewer (GP) may have promoted a social desirability bias from participants. In mitigation, the interview topic guide was developed with several clinicians.

Patient and Public Involvement: None

Results

All participants stressed the importance of patients' LEP and the inadequate provision of interpretation services on care provision. Many commented on how patients were disadvantaged on the grounds of language and how this compromised patient safety. However, unlike other forms of discrimination such as racism, the disadvantage faced by people with LEP was rarely described in terms of discrimination. Instead, staff responded by improvising, using workarounds to get the job done. To illustrate, we present three emergent themes from the data: (i) recognising and resisting discrimination; (ii) the importance of interpretation provision for safe care and; (iii) improvisation around inadequate interpretation

(i) Recognising and resisting discrimination

Healthcare staff readily recognised that people seeking asylum encountered discrimination on the grounds of migration status, nationality, and race:

> *'They will face a lot of discrimination in the quality [of care] … It's been a rude awakening … there's a lot of prejudice and stereotypes of those seeking asylum.'* Participant 7

Many noticed discrimination towards their patients from healthcare staff in other settings such as pharmacy, hospital, and social care. Staff reported inappropriate questioning of patients' entitlement to NHS treatments and social care by service providers, resulting in some being denied care which they had the right to receive. Several portrayed how a dental surgery had refused to see residents from contingency accommodation and labelled this as discriminatory. One participant used these allegations of discrimination as a way of challenging dental surgery staff and advocating for all the residents:

For peer review only - http://bmjopen.bmj.com/site/about/guidelines.xhtml

'I said, "Can I just get this straight? So, you're telling me that one individual was abusive towards your staff, and because of that, you're going to be discriminating against 250 other individuals because you're deciding that everybody who is seeking asylum must be the same and is going to behave like this one person. Is that what you're saying?" … "No, no, no. That's not what we're saying." "Okay, good. Because that would have been discrimination, wouldn't it." These are the conversations that we have to have all of the time.' Participant 1

This illustrates how recognising inequity as discrimination - and articulating it as such - allows individuals to challenge and resist inequitable treatment. Importantly, it demonstrates that some healthcare staff also recognise calling out discrimination as part of their role.

(ii) The importance of interpretation provision for safe care

When asked about wider experiences working in the contingency accommodation, all participants spontaneously stressed the importance of LEP and contrasted it with their previous NHS work. They acknowledged how LEP compounded the multiple complex needs of their asylum-seeking patients and the importance of language interpretation services to reduce inequity:

'I mean, in my view, [translation services] should be a bare minimum for any service delivering health care in the UK ... Because otherwise, you create instant inequalities. Particularly within this very, very vulnerable group of people. Why that isn't a national standard? I don't know.' Participant 1

'It's quite chaotic … The difference, obviously the patient group have a lot of needs that need to be addressed and to be managed, especially the fact that English is not their first language.' Participant 7

Participants frequently associated inadequate interpretation provision with care inequities, even highlighting safety concerns, such as COVID-19 transmission risk. This illustrates the wider public health implications of the derisory provision of information to patients with LEP:

'In the really early days when they were not providing information about COVID in their own languages, a lot of them didn't know what COVID was'. Participant 10

Participant 10 draws attention to the risks posed by medication errors:

'The patients here didn't speak English. They can't read road signs, they don't know where the GP practice is, or the pharmacy is. And when they get to the pharmacy, they can't communicate with the lady behind the desk, so it was really difficult in that sense, and then they would get the medication and be like, "I can't read the label. How do I take this?" And then there are safety

For peer review only - http://bmjopen.bmj.com/site/about/guidelines.xhtml

aspects to that in that they are not ingesting their medication in the right way and things like that, so that was difficult, so things that are normally easy are really, really hard.' Participant 10

This illustrates multiple structural barriers faced by patients with LEP trying to navigate health systems, access care and self-manage. It demonstrates the need for improvements in interpretation services beyond medical consultations, including translation of signage, pamphlets and labels. LEP instigates barriers to accessing safe care and achieving good health not encountered by English speakers, showing how language discrimination propagates existing social hierarchies. Although participants recognised structured and structuring aspects of inadequate interpretation provision, they did not articulate it as discriminatory.

(iii) Improvisation around inadequate interpretation

All participants reported facing challenges accessing adequate interpretation services. Many described the resulting suboptimal care. As part of the pandemic response, volunteers from local refugee charities initially provided face-to-face interpretation at the contingency accommodation for medical consultations. This in-person service was described by Participant 2 as '*so useful … made life so much easier … made such a difference*'. However, this was impossible beyond the first few months as their own voluntary organisations reopened. The clinical team were left using telephone interpretation services, universally reported as problematic and often labelled a *'waste'* of time:

'The waits for [the telephone interpretation service] were quite extensive. 30 minutes, 45 minutes, which had a huge impact on clinical time. And you would often get through and then not be able to get through to the patient. We were wasting a huge number of clinical hours at that point in just trying to get hold of patients and speak to them.' Participant 1

The poor mobile phone signal in the accommodation caused frequent and lengthy interruptions. When staff were able to connect, the quality of interpretation was variable and often substandard:

'Someone would talk at length sometimes and the interpreter would summarise it in a few sentences, so I don't know if I was actually getting the full picture … sometimes because [patients] had partial English they would sometimes say 'I'm not saying that'.' Participant 9

Inadequate interpretation services in secondary care impacted the workload of the primary care participants. Staff in an already-pressured environment took time to fill the gap left by inadequate language provision:

'The doctor sends the referrals, and then the patient gets a letter, but they can't read the letter in English. So, a lot of my job was reading the letter to an interpreter on the phone and explaining it to the patient … Or telephone appointments without times so we can't organise a translator' Participant 2

For peer review only - http://bmjopen.bmj.com/site/about/guidelines.xhtml

Doubts about the quality of interpretation and an imperative to use time efficiently resulted in some participants avoiding telephone interpretation. Instead, they improvised with non-verbal cues, gesticulations and mobile phone apps. This approach is evocative of 'tinkering', described by Mol in 2008 [38], as a way of adjusting towards situationally-determined improved outcomes:

> *'I think I was very much like, I don't want to use this unless I really have to … I tried to communicate like, with facial expressions and hand gestures'* Participant 5

> *'we just didn't have the time [to use telephone interpretation] … we started to use the phone app … I'm sure that it affected care.'* Participant 2

Recognising the importance of good communication for safe care, participants were driven to search for workarounds in the face of limited time, resources and poor-quality interpretation services. Many 'tinkered' to moderate and simplify communication:

> *'it's about how to simplify the language and talk more slowly and talk in shorter sentences because I was going through a translator'* Participant 9

> *'being resourceful, i.e. drawing pictures on paper, using body language or hand gestures as signals and things like that. It is not always easy because of COVID, and you are wearing a face mask … sometimes I use Google Translate or 'Siri' will speak.' Participant 10*

Although some framed this innovation in a positive light, others suggested how this might compromise care:

> *'[some people] they would translate for their friends, which again is an issue with confidentiality and safety at times. I'm not sure if I would want to disclose some things to my friends. That was a last resort … I guess things could have been missed if people didn't want to disclose some information, but that's how we got around it'* Participant 2

Again, the insufficient provision of interpretation was not articulated as discrimination.

For peer review only - http://bmjopen.bmj.com/site/about/guidelines.xhtml

For peer review only

Discussion

This qualitative analysis illustrates how staff understood the disadvantage experienced by those with LEP differently from that based on migration status or ethnicity. When discrimination based on migration status was identified, some staff saw calling it out as part of their role and successfully facilitated change. In contrast, the impact of inadequate interpretation on providing safe and trauma-informed care was not identified as discriminatory, despite all of those interviewed recognising the risks. Consequently, it was not challenged in the same way. Instead, when faced with a lack of available face-to-face interpreters and poor-quality difficult-to-access telephone interpretation, staff 'tinkered', using workarounds such as translation apps, gesticulating or drawings.

The concept of 'tinkering' has been used to explore care practices in various resource-poor contexts.[38,39]. Recently, ethnographic work has demonstrated the resourcefulness of healthcare staff and the necessity of creative innovations in low-income settings: "'[i]f the system doesn't work, you have to make it work for you, for the sake of the patient'".[40] However, tinkering has limits - Reider describes how it can lead to physician disenfranchisement and departure.[41] By framing these practices through the lens of language discrimination, we add to this critical perspective on the practice of tinkering. When staff feel workarounds are their only option, it seems that 'tinkering' can become usual practice, even in resource-rich countries where healthcare faces ideologically driven cuts. Inequalities are thus reproduced, normalised, and embedded within organisational structures and the ability to change discriminatory systems is diminished.

US-based research links the concept of language discrimination and patients' poor experiences of healthcare access.[42,43] However, we appear to be the first to apply the sociological concept of language discrimination to the UK healthcare setting, use it to critically analyse healthcare providers' narratives of care provision, and argue for its more widespread application as a means to address inequalities. In doing so, we make an important theoretical contribution. Empirically, language discrimination is evident throughout this data: differential treatment on the grounds of LEP perpetuates inequalities in care and maintains the normative hierarchies of power and privilege, where people who speak English receive better care. We caution against the assumption that language is a modifiable characteristic and therefore not challenged as readily as other forms of discrimination, such as racism. This speculation overlooks the well-described structural, psychological, social and educational barriers to learning new languages. [44]

Anti-racist scholars assert that naming discrimination and examining how it operates are the first steps in confronting inequitable health systems.[31] We uphold that promoting the application of the concept of language discrimination is the first step in addressing inequitable care for those with LEP. Naming inadequate interpretation provision in healthcare as a form of discrimination works to overcome institutional and social inertia, empowering staff and giving them vocabulary to challenge health inequalities experienced by people with LEP. In the contemporary UK context, the gap in interpretation provision can be understood as part of a broader discriminatory approach to migrants. Technical solutions to improve care are needed, with provision of culturally-sensitive interpreters as the bare minimum.

For peer review only - http://bmjopen.bmj.com/site/about/guidelines.xhtml

Moreover, language discrimination is only one facet of the hostile environment facing migrants in the UK (22). Since undertaking this research, living conditions for people seeking asylum have been further eroded, with plans for offshore processing and accommodation on sea vessels.

Illuminating the issues from a practitioner perspective, this paper contributes to the literature on the underuse of interpreters in healthcare.[14] We emphasise the chasm between academic knowledge, assertions from policymakers, and the reality of conditions facing practitioners. A wealth of literature documents the harms of inadequate interpretation alongside healthcare policy proclaiming that those with LEP should not experience worse care.[16,17] Yet when healthcare staff face high clinical workloads without access to user-friendly high-quality interpretation, using workarounds to get the job done becomes usual practice and inequity becomes institutionalised. It is important to note that in countries where payment is structured differently the extra time required for interpretation may also impact clinician income.

Our empirical findings illuminate why health outcomes are pitiful when provision of interpretation services is inadequate and shed light on the variable uptake of healthcare services across diverse communities. They should interest practitioners and researchers concerned with structural causes of health inequalities. Our analysis highlights that some social groups are underserved rather than 'difficult to reach'. Of relevance for policymakers is the lack of transparency around commissioning for interpretation provision in England and the marked variability in expenditure for comparable demographics.[45,46] A radical overhaul of interpretation provision is crucial to address ethnic inequalities in health.

This paper focuses on language discrimination, a previously under-explored yet important component of ethnic health inequalities. The qualitative data is rich and extensive; however, the study does have limitations. The number of participants is modest due to the specialist nature of the service. We did not collect data about the languages spoken by healthcare staff or how patients' English language proficiency was assessed, both are areas which warrant further investigation. There may be social desirability bias of participants' self-representation to the interviewer, who was a clinician and researcher. These biases could arguably make the findings more salient. The staff at this specialist service often went above and beyond usual care and it is likely that the normalisation and institutionalisation identified here would be readily replicated elsewhere by less committed individuals. Research exploring how these findings translate to other settings would be valuable.

Conclusion

This research provides empirical evidence of how the inadequate provision of interpreters, shown here in the context of the pandemic, appears to force the hand of healthcare staff to use shortcuts. This risks compromising the safe care of people with LEP. Both structural and operational changes are needed to improve healthcare provision for migrants. Inadequate interpretation provision should be understood as part of a complex system of discrimination facing people seeking asylum which requires a radical

For peer review only - http://bmjopen.bmj.com/site/about/guidelines.xhtml

overhaul. More specifically, policymakers must rethink their approach to interpretation provision including training of practitioners to work with interpreters, integrating systems to reduce factors that prevent their use, and prioritising quality over cost to ensure safety.

Endorsement of the concept of language discrimination in healthcare would provide a useful framework for clinicians to better identify and articulate unfair treatment on the grounds of LEP. Borrowing from anti-racist scholarship, we argue that this is the first step in shifting the culture around the ubiquitous but hidden acceptance of a poor standard of care for LEP patients. Staff were tenacious and challenged practices where more overt examples of discrimination were experienced and could be named. Future research should examine healthcare workers' perception of the concept of language discrimination and the utility and limitations of the concept in addressing health inequalities.

CONTRIBUTORSHIP STATEMENT

Louise Tomkow was Principal Investigator of the project. Louise Tomkow and Rebecca Farrington designed the research protocol. Louise Tomkow developed study materials and led ethics application. Gabrielle Prager undertook interviews and coded data. Louise Tomkow and Gabrielle Prager led the analysis. Jessica Drinkwater, Rebecca Farrington and Rebecca Morris undertook analysis. Louise Tomkow led the write up. Louise Tomkow, Gabrielle Prager, Jessica Drinkwater, Rebecca Farrington and Rebecca Morris all contributed to the final manuscript.

COMPETING INTERESTS

None

FUNDING

No specific funding to report for this work. GP is an Academic Clinical Fellow and LT and JD are Clinical Lecturers funded by Health Education England (HEE) and NIHR (grant numbers NA). RM's input was supported by the National Institute for health and care Research (NIHR) Greater Manchester Patient Safety Translational Research Centre (award number PSTRC-2016-003). The views expressed in this publication are those of the author(s) and not necessarily those of the NIHR, NHS or the UK Department of Health and Social Care.

DATA AVAILABILITY STATEMENT

Data contains sensitive information. Please contact lead author to discuss access.

ETHICS APPROVAL STATEMENT

This study involves human participants and was approved by the University of Manchester Research Ethics Committee gave approval (NHS001845).

References

For peer review only

1. Ratna H. The importance of effective communication in healthcare practice. Harvard Public Health Review. 2019 Oct 1;23:1-6.
2. Kang C, Tomkow L, Farrington R. Access to primary health care for asylum seekers and refugees: a qualitative study of service user experiences in the UK. British Journal of General Practice. 2019 Aug 1;69(685):e537-45.
3. White J, Plompen T, Osadnik C, Tao L, Micallef E, Haines T. The experience of interpreter access and language discordant clinical encounters in Australian health care: a mixed methods exploration. International journal for equity in health. 2018 Dec;17(1):1-0.
4. Divi C, Koss RG, Schmaltz SP, Loeb JM. Language proficiency and adverse events in US hospitals: a pilot study. International journal for quality in health care. 2007 Apr 1;19(2):60-7.
5. Flores G, Laws MB, Mayo SJ, Zuckerman B, Abreu M, Medina L, Hardt EJ. Errors in medical interpretation and their potential clinical consequences in pediatric encounters. Pediatrics. 2003 Jan;111(1):6-14.
6. Baker DW, Hayes R, Fortier JP. Interpreter use and satisfaction with interpersonal aspects of care for Spanish-speaking patients. Medical care. 1998 Oct 1:1461-70.
7. Wilson E, Chen AH, Grumbach K, Wang F, Fernandez A. Effects of limited English proficiency and physician language on health care comprehension. Journal of general internal medicine. 2005 Sep;20(9):800-6.
8. Karliner LS, Jacobs EA, Chen AH, Mutha S. Do professional interpreters improve clinical care for patients with limited English proficiency? A systematic review of the literature. Health services research. 2007 Apr;42(2):727-54.
9. Lee JS, Pérez-Stable EJ, Gregorich SE, Crawford MH, Green A, Livaudais-Toman J, Karliner LS. Increased access to professional interpreters in the hospital improves informed consent for patients with limited English proficiency. Journal of general internal medicine. 2017 Aug;32(8):863-70.
10. Jacobs EA, Lauderdale DS, Meltzer D, Shorey JM, Levinson W, Thisted RA. Impact of interpreter services on delivery of health care to limited-English-proficient patients. Journal of general internal medicine. 2001 Jul;16(7):468-74.
11. Karliner LS, Pérez-Stable EJ, Gregorich SE. Convenient access to professional interpreters in the hospital decreases readmission rates and estimated hospital expenditures for patients with limited English proficiency. Medical care. 2017 Mar;55(3):199.
12. John-Baptiste A, Naglie G, Tomlinson G, Alibhai SM, Etchells E, Cheung A, Kapral M, Gold WL, Abrams H, Bacchus M, Krahn M. The effect of English language proficiency on length of stay and in-hospital mortality. Journal of general internal medicine. 2004 Mar;19(3):221-8.
13. Kale E, Syed HR. Language barriers and the use of interpreters in the public health services. A questionnaire-based survey. Patient education and counseling. 2010 Nov 1;81(2):187-91.
14. Diamond LC, Schenker Y, Curry L, Bradley EH, Fernandez A. Getting by: underuse of interpreters by resident physicians. Journal of general internal medicine. 2009 Feb;24(2):256-62.
15. NHS Long term plan, NHE England. Available at: https://www.england.nhs.uk/long-term-plan/ Accessed 13.10.22

For peer review only - http://bmjopen.bmj.com/site/about/guidelines.xhtml

16. Ethnic Inequalities in Healthcare: A Rapid Evidence Review. NHS race and health observatory. Available at: https://www.nhsrho.org/wp-content/uploads/2022/02/RHO-Rapid-Review-Final-Report_Summary_v.4.pdf Accessed 13.10.22
17. Guidance for commissioners interpreting and translation services in primary care, NHS England. Available at: https://www.england.nhs.uk/wp-content/uploads/2018/09/guidance-for-commissioners-interpreting-and-translation-services-in-primary-care.pdf Accessed 13.10.22
18. Patel KC, Hanif W. Ethnic health inequalities in the NHS. BMJ. 2022 Mar 9;376.
19. Barron, D.S., Holterman, C., Shipster, P., Batson, S. and Alam, M., 2010. Seen but not heard–ethnic minorities' views of primary health care interpreting provision: a focus group study. *Primary Health Care Research & Development*, *11*(2), pp.132-141.
20. Gill, P.S., Beavan, J., Calvert, M. and Freemantle, N., 2011. The unmet need for interpreting provision in UK primary care. *PLoS one*, *6*(6), p.e20837.
21. https://www.unhcr.org/uk/asylum-uk
22. Griffiths, M. and Yeo, C., 2021. The UK's hostile environment: Deputising immigration control. *Critical Social Policy*, *41*(4), pp.521-544.
23. Tomkow LJ, Kang CP, Farrington RL, Wiggans RE, Wilson RJ, Pushkar P, Tickell-Painter MC, Lee AR, Whitehouse ER, Mahmood NG, Lawton KM. Healthcare access for asylum seekers and refugees in England: a mixed methods study exploring service users' and health care professionals' awareness. European Journal of Public Health. 2020 Jun 1;30(3):527-32.
24. Patel P, Bernays S, Dolan H, Muscat DM, Trevena L. Communication experiences in primary healthcare with refugees and asylum seekers: a literature review and narrative synthesis. International journal of environmental research and public health. 2021 Feb;18(4):1469.
25. Arnold FW. How can we help asylum seekers get better healthcare?. bmj. 2022 Feb 7;376.
26. The Home Office. The use of temporary hotels to house asylum seekers during Covid-19. August 2020. Available at: https://homeofficemedia.blog.gov.uk/2020/08/08/the-use-of-temporary-hotels-to-house-asylum-seekers-during-covid-19/ Accessed August 2022
27. Jones L, Phillimore J, Fu L, Hourani J, Lessard-Phillips L, Tatem B. Doctors of the World Report. *They just left me Asylum seekers, health, and access to healthcare in initial and contingency accommodation. 2022.* Report. Available at: https://www.doctorsoftheworld.org.uk/wp-content/uploads/2022/04/DOTW-Access-to-healthcare-in-initial-and-contingency-accommodation-report-April-2022.pdf. Accessed March 2023
28. Written evidence submitted by Doctors of the World (DOTW) (COR0203) [Internet]. [cited 2022 Mar 28]. Available from: https://bjgp.org/content/69/685/e537#ref-6
29. Farrant O, Eisen S, Van Tulleken C, Ward A, Longley N. Why asylum seekers deserve better healthcare, and how we can give it to them. bmj. 2022 Jan 6;376.
30. Lippi-Green R. English with an accent: Language, ideology, and discrimination in the United States. Routledge; 2012 Mar 15.
31. Jones CP. Confronting institutionalized racism. Phylon (1960-). 2002 Jan 1:7-22.
32. Lee JJ, Rice C. Welcome to America? International student perceptions of discrimination. Higher education. 2007 Mar;53(3):381-409.

For peer review only - http://bmjopen.bmj.com/site/about/guidelines.xhtml

33. Yoo HC, Gee GC, Takeuchi D. Discrimination and health among Asian American immigrants: Disentangling racial from language discrimination. Social science & medicine. 2009 Feb 1;68(4):726-32.
34. Language requirements can be a legal minefield. Employment Law. Available at:
35. https://www.peoplemanagement.co.uk/article/1745542/language-requirements-legal-minefield Accessed 13.10.22
36. Core20PLUS5 – An approach to reducing health inequalities. Available at: https://www.england.nhs.uk/about/equality/equality-hub/national-healthcare-inequalities-improvement-programme/core20plus5/#:~:text=Core20PLUS5%20is%20a%20national%20NHS,clinical%20areas%20requiring%20accelerated%20improvement Accessed 9.11.22
37. Tomkow, L., Prager, G., Worthing, K. and Farrington, R., 2023. In critique of moral resilience: UK healthcare professionals' experiences working with asylum applicants housed in contingency accommodation during the COVID-19 pandemic. *Journal of Medical Ethics*.
38. Mol, A., 2008. *The logic of care: Health and the problem of patient choice*. Routledge.
39. Trundle, C., 2020. Tinkering care, state responsibility, and abandonment: nuclear test veterans and the mismatched temporalities of justice in claims for health care. *Anthropology and Humanism*, *45*(2), pp.202-211.
40. Vernooij, E., Koker, F. and Street, A., 2022. Responsibility, repair and care in Sierra Leone's health system. *Social Science & Medicine*, *300*, p.114260.
41. Rieder, S., 2017. Tinkering toward departure: The limits of improvisation in rural Ethiopian biomedical practices. *Social Science & Medicine*, *179*, pp.1-8.
42. Weng, S.S. and Wolfe III, W.T., 2016. Asian American health inequities: An exploration of cultural and language incongruity and discrimination in accessing and utilizing the healthcare system. *International Public Health Journal*, *8*(2).
43. Halim, M.L., Moy, K.H. and Yoshikawa, H., 2017. Perceived ethnic and language-based discrimination and Latina immigrant women's health. *Journal of Health Psychology*, *22*(1), pp.68-78.
44. Thomas DR. A general inductive approach for analyzing qualitative evaluation data. American journal of evaluation. 2006 Jun;27(2):237-46.
45. Morrice L, Tip LK, Collyer M, Brown R. 'You can't have a good integration when you don't have a good communication': English-language learning among resettled refugees in England. Journal of Refugee Studies. 2021 Jun 26;34(1):681-99.
46. Lehane D, Oliver P, Mitchell C, Burton C. Interpreter costs across clinical commissioning groups in England 2017–2018: a cross sectional survey using 'freedom of information'requests. British Journal of General Practice. 2020 Jun 1;70(suppl 1).

For peer review only - http://bmjopen.bmj.com/site/about/guidelines.xhtml

Standards for Reporting Qualitative Research (SRQR)*

http://www.equator-network.org/reporting-guidelines/srqr/

Page/line no(s).

Title and abstract

**Title** - Concise description of the nature and topic of the study Identifying the study as qualitative or indicating the approach (e.g., ethnography, grounded theory) or data collection methods (e.g., interview, focus group) is recommended	Page 1 Line 1
**Abstract** - Summary of key elements of the study using the abstract format of the intended publication; typically includes background, purpose, methods, results, and conclusions	Page 1 lines 3 onwards

Introduction

**Problem formulation** - Description and significance of the problem/phenomenon studied; review of relevant theory and empirical work; problem statement	Pages 3-5
**Purpose or research question** - Purpose of the study and specific objectives or questions	Pages 3-5

Methods

**Qualitative approach and research paradigm** - Qualitative approach (e.g., ethnography, grounded theory, case study, phenomenology, narrative research) and guiding theory if appropriate; identifying the research paradigm (e.g., postpositivist, constructivist/ interpretivist) is also recommended; rationale**	Pages 5-6
**Researcher characteristics and reflexivity** - Researchers' characteristics that may influence the research, including personal attributes, qualifications/experience, relationship with participants, assumptions, and/or presuppositions; potential or actual interaction between researchers' characteristics and the research questions, approach, methods, results, and/or transferability	Page 6
**Context** - Setting/site and salient contextual factors; rationale**	Pages 3, 5-6
**Sampling strategy** - How and why research participants, documents, or events were selected; criteria for deciding when no further sampling was necessary (e.g., sampling saturation); rationale**	Page 5
**Ethical issues pertaining to human subjects** - Documentation of approval by an appropriate ethics review board and participant consent, or explanation for lack thereof; other confidentiality and data security issues	Page 6
**Data collection methods** - Types of data collected; details of data collection procedures including (as appropriate) start and stop dates of data collection and analysis, iterative process, triangulation of sources/methods, and modification of procedures in response to evolving study findings; rationale**	Pages 5-6

For peer review only - http://bmjopen.bmj.com/site/about/guidelines.xhtml

**Data collection instruments and technologies** - Description of instruments (e.g., interview guides, questionnaires) and devices (e.g., audio recorders) used for data collection; if/how the instrument(s) changed over the course of the study	Pages 5-6
**Units of study** - Number and relevant characteristics of participants, documents, or events included in the study; level of participation (could be reported in results)	Pages 5-6
**Data processing** - Methods for processing data prior to and during analysis, including transcription, data entry, data management and security, verification of data integrity, data coding, and anonymization/de-identification of excerpts	Pages 5-6
**Data analysis** - Process by which inferences, themes, etc., were identified and developed, including the researchers involved in data analysis; usually references a specific paradigm or approach; rationale**	Pages 5-6
**Techniques to enhance trustworthiness** - Techniques to enhance trustworthiness and credibility of data analysis (e.g., member checking, audit trail, triangulation); rationale**	Pages 5-6

Results/findings

**Synthesis and interpretation** - Main findings (e.g., interpretations, inferences, and themes); might include development of a theory or model, or integration with prior research or theory	Pages 6-11
**Links to empirical data** - Evidence (e.g., quotes, field notes, text excerpts, photographs) to substantiate analytic findings	Pages 6-11

Discussion

**Integration with prior work, implications, transferability, and contribution(s) to the field -** Short summary of main findings; explanation of how findings and conclusions connect to, support, elaborate on, or challenge conclusions of earlier scholarship; discussion of scope of application/generalizability; identification of unique contribution(s) to scholarship in a discipline or field	Pages 12-14
**Limitations** - Trustworthiness and limitations of findings	Page 13

Other

**Conflicts of interest** - Potential sources of influence or perceived influence on study conduct and conclusions; how these were managed	Page 17
**Funding** - Sources of funding and other support; role of funders in data collection, interpretation, and reporting	Page 17

*The authors created the SRQR by searching the literature to identify guidelines, reporting standards, and critical appraisal criteria for qualitative research; reviewing the reference lists of retrieved sources; and contacting experts to gain feedback. The SRQR aims to improve the transparency of all aspects of qualitative research by providing clear standards for reporting qualitative research.

For peer review only

**The rationale should briefly discuss the justification for choosing that theory, approach, method, or technique rather than other options available, the assumptions and limitations implicit in those choices, and how those choices influence study conclusions and transferability. As appropriate, the rationale for several items might be discussed together.

<u>Reference:</u>

O'Brien BC, Harris IB, Beckman TJ, Reed DA, Cook DA. **Standards for reporting qualitative research: a synthesis of recommendations.** *Academic Medicine*, Vol. 89, No. 9 / Sept 2014 DOI: 10.1097/ACM.0000000000000388

For peer review only

For peer review only - http://bmjopen.bmj.com/site/about/guidelines.xhtml
